# Novel *neo*-clerodane diterpenoids from *Teucrium quadrifarium* and their anti-ferroptosis effect

**DOI:** 10.1007/s13659-024-00489-1

**Published:** 2025-01-06

**Authors:** Huan Wang, Han-Fei Liu, Xiao-Qiao Yang, Yu-Qiong Liao, Fen-Cong Pan, Jin-Yu Li, Hua-Yong Lou, Wei-Dong Pan

**Affiliations:** 1 State Key Laboratory of Functions and Applications of Medicinal Plants, Guizhou Medical University, Guiyang, 550014 People’s Republic of China; 2 Natural Products Research Center of Guizhou Province, Guiyang, 550014 People’s Republic of China

**Keywords:** *Teucrium quadrifarium*, Lamiaceae, *Neo-*clerodane, Ferroptosis inhibitory activity, ROS

## Abstract

Teucrifarides A–D (**1**–**4**), four previously unreported *neo*-clerodane-type diterpenoids, combined with sixteen known analogs (5–20), were purified from *Teucrium quadrifarium.* The absolute forma of compounds **1**–**4** were determined via spectroscopic and ECD calculation methods, together with X-ray crystallography experiments. Among them, compound **1** possessed a 5,20-epoxy ring featuring a unique cage-like 12-oxatricyclo [5.3.2.0^1,6^]undecane skeleton. Meanwhile, 2 incorporated a 6,20-epoxy ring with a novel 12-oxatricyclo [6.2.2.0^2,7^]undecane skeleton. Compounds **1** and **12** exhibited significant inhibitory effects against HT-22 cells ferroptosis induced by RSL3, with EC_50_ values of 11.8 ± 1.0 μM, and 4.52 ± 1.24 μM, respectively. Moreover, ROS accumulation in HT22 cells treated with compound **1** was also observed.

**Graphical Abstract:**

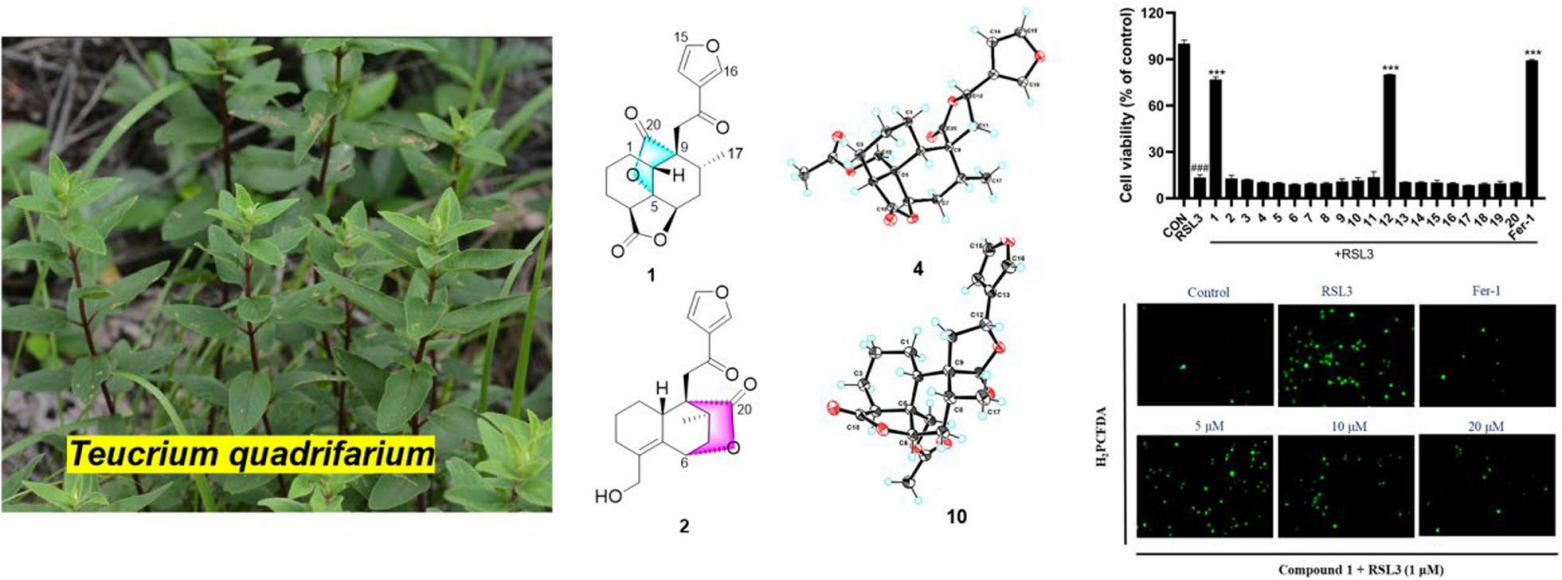

**Supplementary Information:**

The online version contains supplementary material available at 10.1007/s13659-024-00489-1.

## Introduction

The genus *Teucrium* (Lamiaceae) comprises shrubs and herbs, and the majority predominantly distributed in southwest regions of China [1]. The phytochemical research on this genus revealed that diterpenes, flavonoids, and triterpenes were the main metabolites of this plant [2]. Diterpenoids are well known as one of the primary secondary metabolites within the Lamiaceae family, and several complex diterpenoid scaffolds have been reported from the genus *Teucrium* [3–5]. Especially, abietane and clerodane exhibit interesting biological activities, including anti-ferroptosis [6], anti-inflammatory [7], anti-neuroinflammation [8], antifeedant [9], and cytotoxic activities [10], which have drawn the interest of pharmacologist.

*Teucrium quadrifarium*, a plant of the genus *Teucrium*, is a folk medicine to treat wind heat syndrome, and nosotoxicosis [11, 12]. However, to date, the phytochemical investigation of *T. quadrifarium* has yielded only a few natural compounds, such as flavonoids [13] and *neo*-clerodane diterpenoids [14]. As part of our efforts to obtain structurally intriguing and biologically significant clerodane-diterpenoids, a phytochemical study of *T. quadrifarium* was undertaken. As a result, twenty clerodane diterpenoids, including four previously undescribed *neo-*clerodane diterpenoids (**1**–**4**) were obtained. Herein, the structural elucidation, and anti-ferroptosis effects of these isolates were reported.

## Results and discussion

**Fig. 1:**
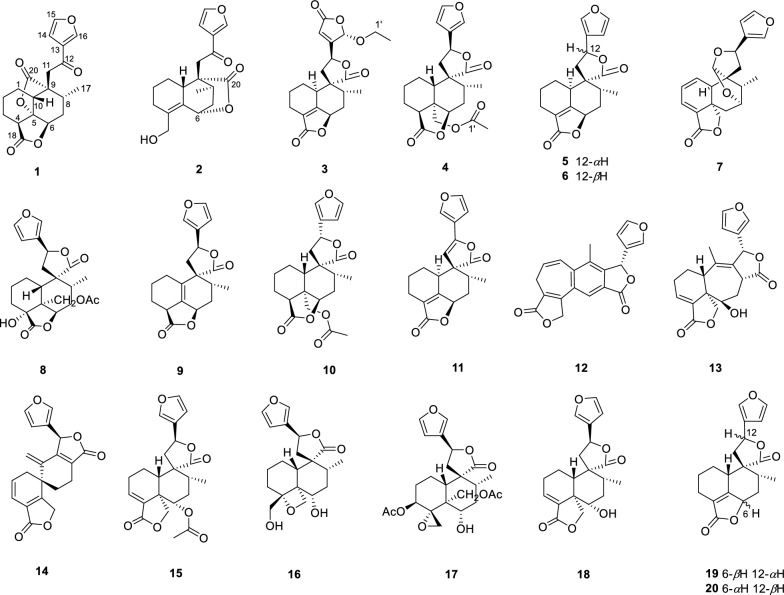
Key ^1^H-^1^H COSY and HMBC correlations of compounds **1**–**4**

Teucrifaride A (**1**) possesses the molecular formula of C_19_H_20_O_6_, based on the HR-ESI-MS ion at *m/z* 367.1149 [M + Na]^+^ (cacld 367.1152). In the ^1^H NMR spectrum (Table 1), one methyl (*δ*_H_ 1.03) and three olefinic protons (*δ*_H_ 6.75, 7.46, 8.09) were observed. Moreover, in the ^13^C NMR (Table 2) spectrum, 19 carbon resonances were assigned to one methyl, five methylenes, seven methines, and six non-protonated carbons, respectively. Moreover, a characteristic furan ring [*δ*_H_/*δ*_C_ 6.75 (d, *J* = 2.4 Hz)/108.4 (C-14), 7.46 (t, *J* = 1.8 Hz)/144.7 (C-15), 8.09 (s)/147.6 (C-16), and *δ*_C_ 127.8 (C-13)] was deduced based on the 1D NMR data. The ^1^H-^1^H COSY correlations of H_2_−1/H_2_−2/H_2_−3/H-4, H-10/H_2_−1, and H-6/H_2_−7/H-8, H-8/H_3_−17, combined with the HMBCs of H-4 (*δ*_H_ 3.12) with C-5, C-18, of H-6 (*δ*_H_ 4.50) with C-5, C-18, of H-10 (*δ*_H_ 2.90) with C-6, C-8, C-11, C-20, of H_3_−17 (*δ*_H_ 1.03) with C-9, and of H-11, H-13, and H-16 with C-12 (*δ*_C_ 191.8) suggested that compound **1** was a characteristic *neo*-clerodane diterpenoid analogue, with one *γ*-lactonic ring unit [15]. In addition, an additional *γ*-lactonic ring fragment constructed via C-5–C-20 according to the key HMBC correlations of H-10, H-8, and H-11 with C-20, combined with the downfield characteristic at C-5 (*δ*_C_ 83.9). Thus, as shown in Fig. 1, the planner structure of **1** was constructed.

**Table 1:** ^1^H NMR (600 MHz) data of compounds **1**–**4** in CDCl_3_ (*δ* in ppm, *J* in Hz)

Position	1	2	3	4
	*δ*_H mult._ (*J* in Hz)	*δ*_H mult._ (*J* in Hz)	*δ*_H mult._ (*J* in Hz)	*δ*_H mult._ (*J* in Hz)
1*α*	1.59 m	0.94 m	1.31 m	1.56 m
1*β*	0.99 m	1.65 m	1.99 m	1.89^a^
2*α*	1.74 m	1.58 m	1.60 m	1.53 m
2*β*	1.23 dt (13.8, 3.0)	1.83 m	1.97 m	1.89^a^
3*α*	2.23^a^	2.15 m	2.18 m	1.72 m
3*β*	1.62 m	2.25 m	2.34 m	2.03 m
4	3.12 d (6.6)	–	–	2.86 dd (3.0, 7.2)
6	4.50 t (7.8)	5.41 d (4.8)	4.99 m	4.48 t (3.0)
7	2.08 ddd (15.0, 7.8, 2.4)	1.72 dt (13.8, 4.2)	1.41 td (12.6, 4.2)	2.06 m
7*β*	2.23^a^	2.23 m	2.36 m	2.37 m
8	2.46 m	2.91 m	2.22 m	1.92 m
10	2.90 dd (12.6, 4.8)	3.04 m	3.23 m	1.76 dd (12.0, 1.8)
11a	3.31 d (18.6)	3.24 q	1.97 m	2.32 dd (13.8, 7.2)
11b	2.86 d (18.6)	–	2.51 dd (14.4, 9.6)	2.44 dd (13.8, 9.6)
12	–	–	5.26 t (8.4)	5.33 dd (10.2, 7.8)
14	6.75 d (2.4)	6.76 dd (1.8, 1.2)	6.09 s	6.38 d (1.2)
15	7.46 t (1.8)	7.44 t (1.8)	–	7.44 t (1.8)
16	8.09 s	8.14 t (1.2)	5.89 s	7.45 s
17	1.03 d (7.8)	0.91 d (7.2)	1.37 d (7.8)	1.06 d (6.6)
18a	–	4.10 d (8.0)	–	–
18b	–	4.18 d (8.0)	–	–
19a	–	–	–	4.45 d (12.6)
19b	–	–	–	4.86 d (12.6)
1′a	–	–	3.78 m	–
1ʹb	–	–	3.99 m	–
2′	–	–	1.26 t (7.2)	2.09 s

^a^Overlapped signal

**Table 2:** ^13^C NMR (150 MHz) data of compounds **1**–**4** in CDCl_3_

Position	1	2	3	4
1	21.9	24.9	23.4	21.3
2	20.9	21.8	21.4	24.2
3	20.1	26.7	20.2	19.5
4	40.8	133.5	128.3	45.4
5	83.9	133.2	161.6	46.0
6	76.2	73.5	76.0	77.7
7	30.9	32.9	35.9	29.2
8	31.5	31.7	38.3	34.4
9	51.2	50.2	51.4	49.9
10	41.0	41.4	36.3	46.9
11	37.9	36.7	37.5	41.8
12	191.8	192.8	72.0	72.0
13	127.8	128.1	163.3	124.8
14	108.4	108.5	119.0	108.1
15	144.7	144.5	169.1	144.4
16	147.6	147.5	101.9	140.0
17	18.1	19.9	14.1	16.3
18	173.3	61.6	172.5	177.2
19				59.3
20	177.3	176.1	177.1	177.7
1′			67.4	170.5
2′			15.2	21.0

**Fig. 2:**
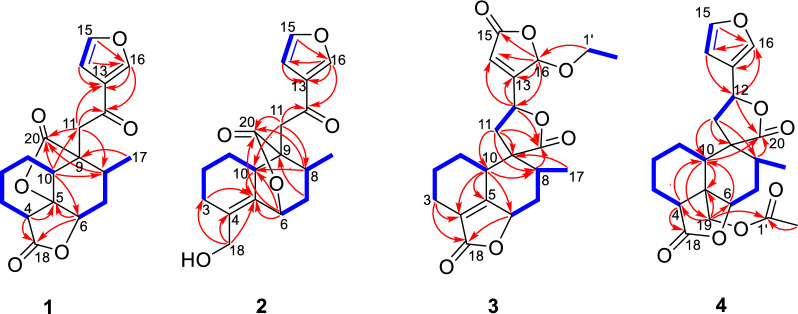
Key NOESY correlations of compounds **1**–**4**

**Fig. 3:**
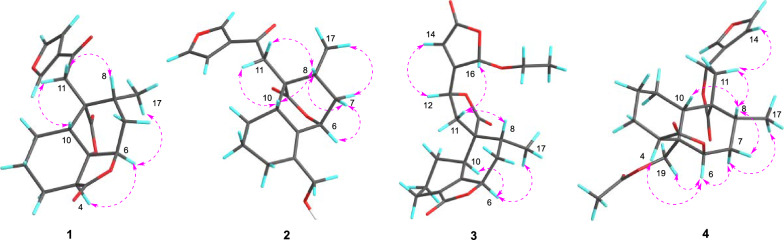
Experimental and Calculated ECD curves of **1**–**3**

The NOESY data was used for analyzing the relative configuration of **1** (Fig. 2). The signals of CH_3_−17/H-6/H-4, assigned these groups were as *α*-orientation. On the contrary, the correlations of H-8/H-10/H-11 demonstrated these protons were *β*-orientation. Ultimately, the structure of **1** was fixed to be 4*R*,5*R*,6*S*,8*S*,9*S*, and 10*S*, through ECD calculation method (Fig. 3).

Teucrifaride B (**2**) with a molecular formula C_19_H_22_O_5_ in line with its HR-ESI-MS ion at *m/z* 353.1346 [M + Na]^+^ (cacld 353.1359). The NMR data of **2** closely resembled those of **1**, indicated these two compounds share the almost same *neo*-clerodane skeleton. The main difference was a methylene [*δ*_H_ 4.14 (H-18), *δ*_C_ 61.6 (C-18)] in **2** replace of a carbonyl carbon *δ*_C_ 173.3 (C-18) in **1**. Besides, an unusual *δ*-lactone ring was formed via C-6–C-7–C-8–C-9–C-20 based on the HMBC correlations of H-6, H-8, H-10, and H-11 with C-20, combined with the signals of H-6/H_2_−7/H-8/CH_3_−17 in the COSY spectrum (Fig. 1). Therefore, the planner structure of **2** was elucidated. Furthermore, the NOESY correlations (Fig. 2) of H-10/H-8/H-11, and H-8/H-7*β*/H-6, manifested that H-10, H-8, H-6, and H-11 were *β*-orientation. Similarly, CH_3_−17 was *α*-oriented deduced by the NOESY signals of CH_3_−17/H-7*α*. Subsequently, the structure of **2** was determined to be 6*R*, 8*S*, 9*S*, 10*R*, according to the calculated ECD curve was the same with that of experimental ECD curves in the range of 200–400 nm (Fig. 3).

**Fig. 4:**
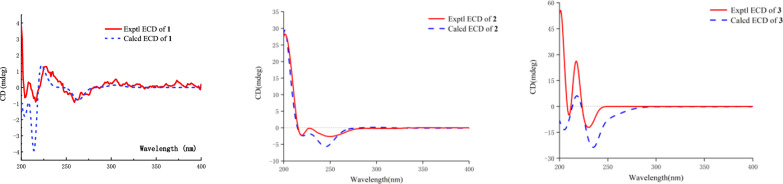
The chemical structures of compounds **1**–**20**

Teucrifaride C (**3**) was yield as a white powder. The HR-ESI-MS data (*m/z* 411.1408 [M + Na]^+^; cacld for 411.1414) suggested a molecular of C_21_H_24_O_7_. By comparison of 1D NMR data, the structure of **3** is similar to the structure of teucvidin (**5**) [16], except for an ethoxy group [*δ*_H_ (1.26, t, *J*** = **7.2 Hz), 3.78(1H, m), 3.99 (1H, m); *δ*_C_ 15.2, 67.4] and an *α*,*β*-unsaturated-*γ*-lactone fragment (*δ*_C_ 169.1, 163.3, 119.0, 101.9) in **3**. Furthermore, the ethoxy unit was fixed at C-16 due to the ^1^H-^1^H COSY correlations of H_3_−2′/H_2_−1ʹ (Fig. 1), together with the HMBC correlations (Fig. 1) from H_2_−1′ (*δ*_H_ 3.78, 3.99) to C-16. Besides, the HMBC correlations from H-16 to C-15, C-13, C-14, and from H-12 to C-13, C-14, C-16, further constructed an *a*,*b*- unsaturated-*γ*-lactone fragment at C-12. Furthermore, the signals of H-10/H-6/H_3_−17 in the NOESY spectrum revealed that they were *α*-oriented. In contrast, the NOESY correlations of H-8/H-11b/H-16, and H-12/H-14 were deserved indicating that H-8, H-16 were *β*-oriented (Fig. 1). The test ECD curve of **3** was well matched with that of calculated ECD spectra (Fig. 3), and the structure of **3** was assigned as Fig. 4.

**Fig. 5:**
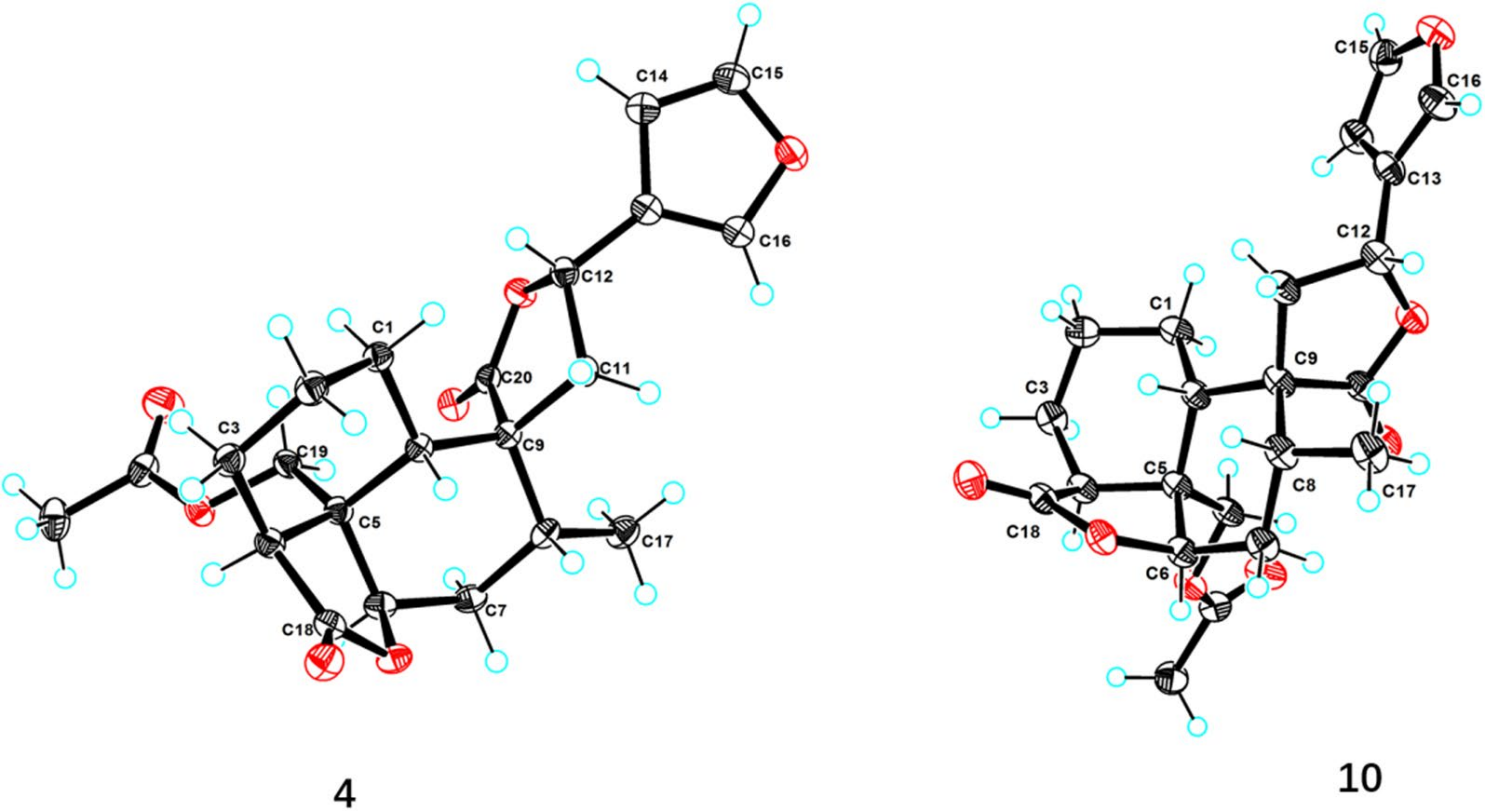
X-ray ORTEP drawing of compounds **4** and **10**

Teucrifaride D (**4**), a colorless acicular crystal, with the molecular formula of C_22_H_26_O_7_ based on the HR-ESI-MS ion at *m/z* of 425.1559 [M + Na]^+^ (C_22_H_26_O_7_Na, cacld for 425.1571) and ^13^C NMR data. After careful analysis of 1D NMR data of **4** (Tables 1 and 2), we deduced that **4** possesses the same structure as that of teucvisin B (**10**) [17]. Moreover, the NOESY correlations of H-10/H-7*β*/H-8/H-11/H-14, combined with the opposite signals of H-6/H-7*α*/CH_3_−17, further suggested H-12 was *α-*oriented. Finally, the structure of **4** was undoubtedly confirmed to be 4*S*,5*R*,6*R*,8*R*,9*R*,10*S*,12*R* through the signal-crystal X-ray diffraction experiment (Fig. 5).

Sixteen known diterpenoids were determined to be teucvidin (**5**) [16], 12-epiteucvidin (**6**) [16], salvifaricin (**7**) [18], 19-acetyl-teuspinin (**8**) [19], isoteuflin (**9**) [20], teucvisin B (**10**) [17], crotocaudin (**11**) [21], isosalvipuberulin (**12**) [22], de-*O*-acetylsalvigenolide (**13**) [23], salvileucalin A (**14**) [18], 6*a*-acetoxyteuscordin (**15**) [24], montanin D (**16**) [25], teumicropodin (**17**) [26], 6-*a*-hydroxy-teuscordin (**18**) [27], teucvin (**19**) [28] and 12-epiteuflin (**20**) [16] through comparison of the NMR data with those of literatures.

**Fig. 6:**
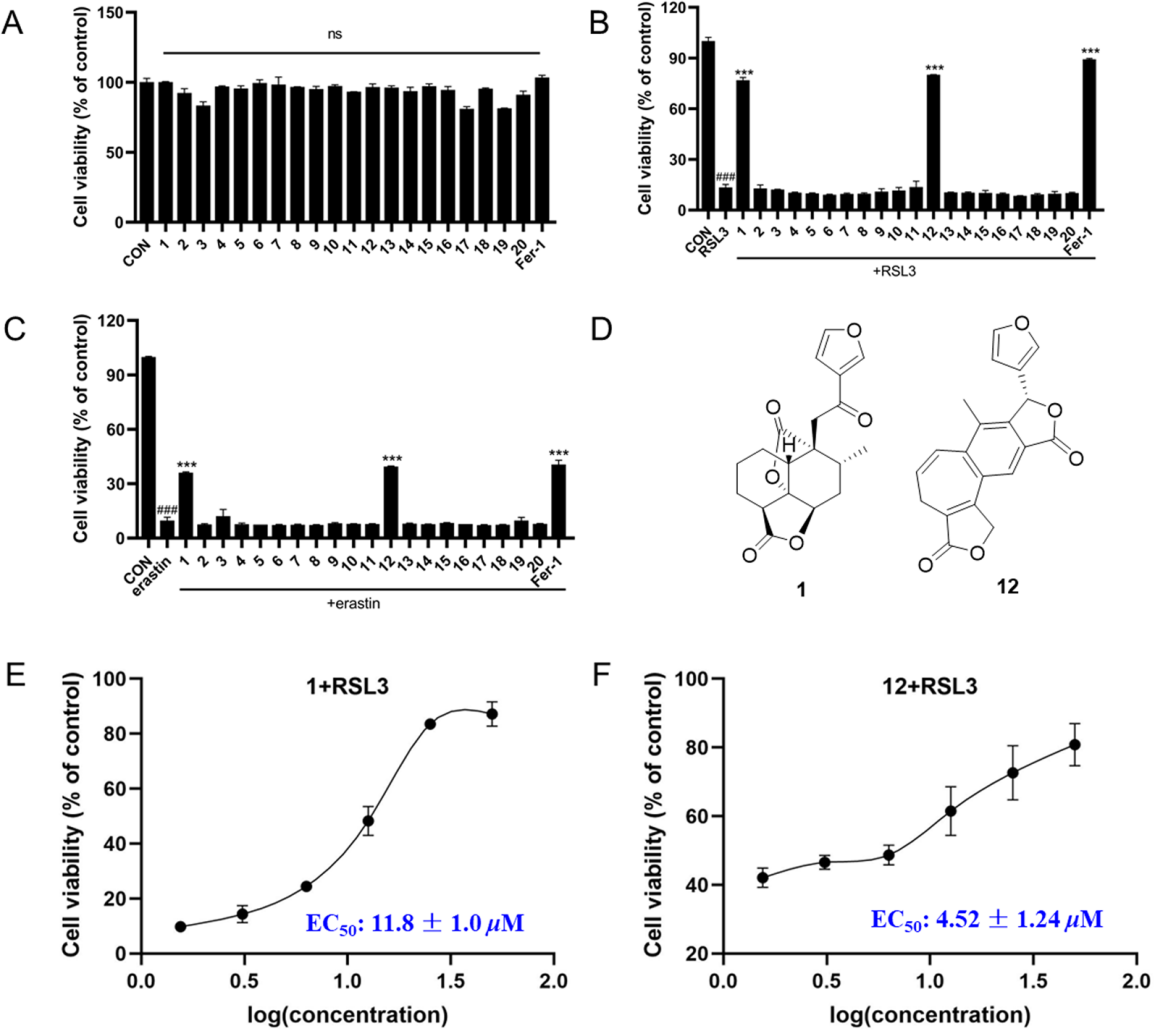
Effects of compounds **1**–**20** on RSL3-induced HT22 cells ferroptosis. **A** Cell viability of HT22 cells treated with 40 μM isolated compounds and 1 μM Fer-1. **B** Cell viability of HT22 cells co-treated with 1 μM RSL3 and 40 μM isolated compounds and 1 μM Fer-1 for 24 h determined by an MTT assay. **C** Cell viability of HT22 cells co-treated with 1 μM erastin and 40 μM isolated compounds and 1 μM Fer-1 for 24 h determined by an MTT assay. **D** Chemical structures of **1** and **12**. Dose–response relationship of compounds **1** (E), and **12** (F) inhibiting RSL3-induced ferroptosis in the HT22 cells. Data represent the means ± SEM from triplicate experiments. ^###^*P* < 0.0001 compared with control cell; ^***^P < 0.0001 compared with cells treated with RSL3

Considering that clerodane-diterpenoids were reported a remarkable anti-ferroptosis effect [6, 29], we evaluated the ferroptosis inhibitory effect of all isolates in HT22 cells. Firstly, their cytotoxicity was assayed, and none of them exhibited cytotoxicity on HT22 cells (Fig. 6A) at 40 μM. Then, the anti-ferroptosis effect of the isolates on HT22 cells deduced by RSL3 and erastin as depicted in Fig. 6B and 6C. Notably, of them, compounds **1** and **12** indicated obvious inhibitory effect against RSL3-induced ferroptosis, and potent ferroptosis inhibition on erastin-induced model, respectively. And the EC_50_ values of **1** and **12** were 11.8 ± 1.0 μM, and 4.52 ± 1.24 μM, respectively, for above two different induced-models (Fig. 6D).

**Fig. 7:**
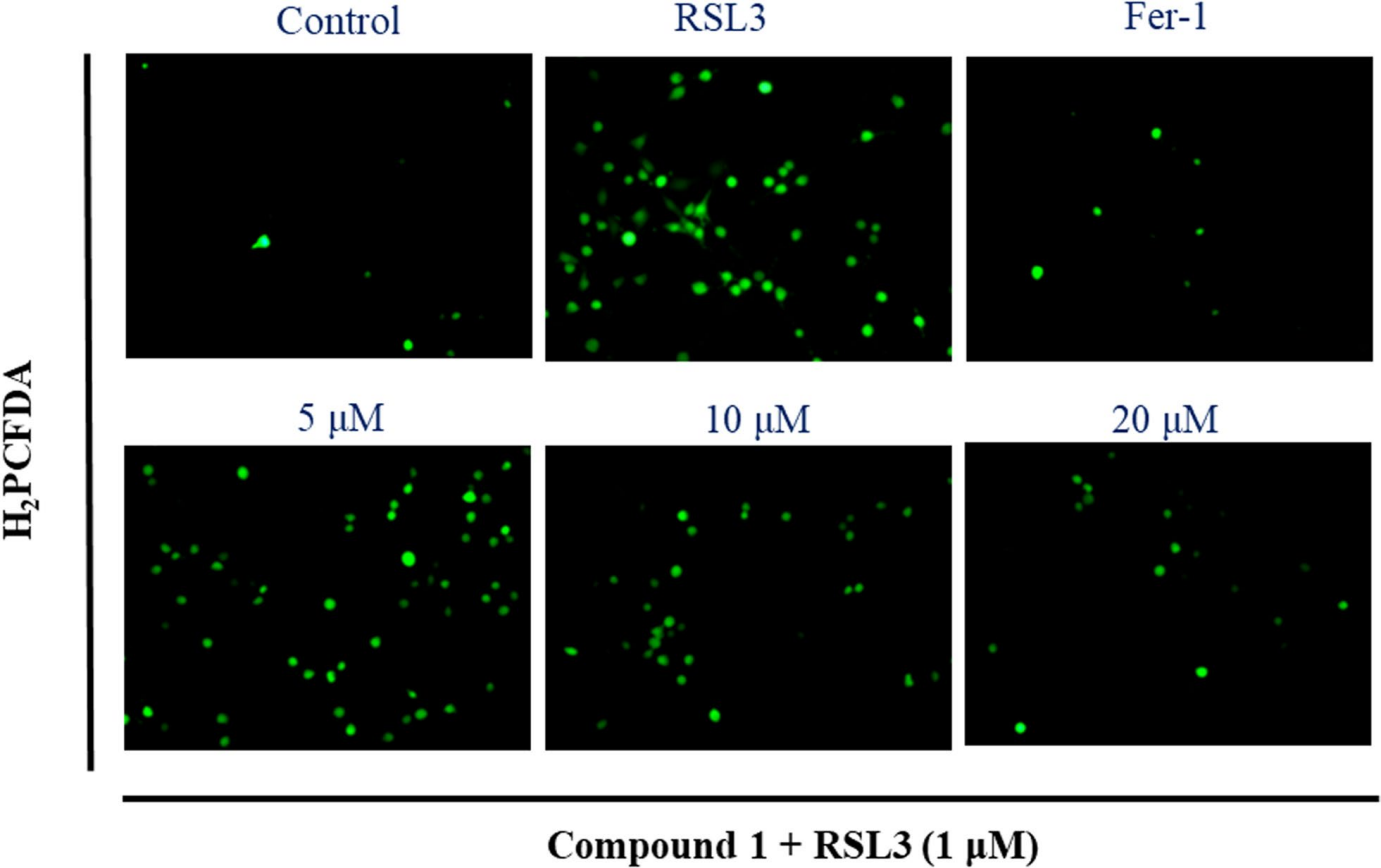
The intracellular ROS levels was detected by fluorescence microscopy: HT22 cells induced by RSL3 and treated with an indicated concentrations of compound **1** (5, 10, and 20 μM) or Fer-1(1 μM) for 4 h under a fluorescence microscope after H2PCFDA staining (20x)

The increase of intracellular reactive oxygen species (ROS) was characteristic biomarkers of ferroptosis. To further explore the ferroptosis inhibition of new compound **1** against RSL3-reduced HT22 cells, the intracellular ROS levels by fluorescence microscopy was detected. As shown in Fig. 7, at the concentrations of 5, 10, and 20 μM, compound **1** can prominently inhibit intracellular ROS accumulation in a dose-dependent manner. The above results preliminarily uncovered that new compound **1** exerts anti-ferroptosis activity should be related to target mitochondria. However, to date, there is no clear mechanism for ferroptosis. Thus, compound **1** should be further researched for its anti-ferroptosis effective mechanism in the future.

## Conclusion

In conclusion, twenty *neo*-clerodane isloates were isolated from *T. quadrifarium*, including four new (**1**–**4**) and sixteen known ones (**5**–**20**). Among them, fifteen compounds were isolated from this plant for the first time, and the results enriches the chemical components of this plant. Additionally, new compound **1** exhibited a significant ferroptosis inhibitory effect against RSL3-induced HT22 cells.

## Experimental section

### General experimental procedures

XtaLAB Pro diffractometer was used to determine X-ray crystallography. HR-ESI–MS, IR, UV, NMR spectra were determined as described previously [30].

### Plant material

The whole plants of *T. quadrifarium* were collected in Pingtang County, Guizhou Province, China, in August, 2021, and identified by Prof. Ni Zhang. A specimen voucher (No. TZC20210806) is stored in the Natural Products Research Center of Guizhou Province.

### Extraction and isolation

The air-dried material (20.0 kg) was smashed and extracted with 95% aqueous EtOH (3 × 70 L, each 2 h) under reflux. The crude EtOH extract was obtained by evaporation under vacuum conditions. The crude samples were suspended in H_2_O and then leached with petroleum ether (PE), and ethyl acetate (EA), respectively. The PE part (1047.4 g) and EA part (819.9 g) were evaporated to dryness. The PE layer was purified with silica gel chromatography, employing stepwise gradient elution with PE/EA mixture ranging from.

100:0 to 0:100, to afford seven fractions (Fr. A–G), according to TLC plates. Fr. D (74.5 g) was isolated on CC (MeOH/H_2_O, from 50:50 to 95:5) to yield eight fractions (Fr. D1–D8). Fr. D5 (13.0 g) was further purified via Sephadex LH-20 (CH_2_Cl_2_/MeOH, 1:1) to yield compound **5** (32.3 mg), 6 (18.4 mg). Compound **1** (MeOH/H_2_O, 57:43, *t*_R_ 19.5 min, 9.3 mg), and 9 (MeCN/H_2_O, 49:51, *t*_R_ 26.0 min, 9.1 mg) were obtained from Fr. D6 via semi-preparative HPLC. And compounds **4** (MeCN/H_2_O, 45:55, *t*_R_ 53.0 min, 5.6 mg), **10** (MeCN/H_2_O, 45:55, *t*_R_ 47.0 min, 3.6 mg) isolated from Fr. D6 by semi–preparative HPLC. Fr. D4 was isolated through Sephadex LH-20 (CH_2_Cl_2_/MeOH, 1:1) and semi-preparative HPLC to give compounds **11** (MeOH/H_2_O, 64:36, *t*_R_ 27.5 min, 9.6 mg), and **12** (MeOH/H_2_O, 48:52, *t*_R_ 31.0 min, 1.9 mg). Fr. D3 was repeatedly separated on CC, and subsequently purified to have **7** (13.2 mg) and **8** (6.3 mg) via Sephadex LH-20 (CH_2_Cl_2_/MeOH, 1:1). Fr. F (87.5 g) was chromatographed on CC (MeOH/H_2_O, from 30:70 to 95:5) to give eight fractions (Fr. F1–Fr. F8). Fr. F2 (17.2 g) further afforded six fractions (Fr. F2a–Fr. F2f) after purified by silica gel (CH_2_Cl_2_/MeOH, from 100:1 to 0:100). Compounds **13** (MeCN/H_2_O, 35:65, *t*_R_ 27.0 min, 2.8 mg), **14** (MeCN/H_2_O, 35:65, *t*_R_ 40.0 min, 3.3 mg), and **15** (MeOH/H_2_O, 48:52, *t*_R_ 48.0 min, 17.9 mg) were obtained from Fr. F2c, and compounds **16** (MeCN/H_2_O, 40:60, *t*_R_ 36.0 min, 17.8 mg), **17** (MeOH/H_2_O, 48:52, *t*_R_ 39.0 min, 12.5 mg), **18** (MeCN/H_2_O, 48:52, *t*_R_ 26.0 min, 9.8 mg) were yield from Fr.F2d, by semi-preparative HPLC.

The EA layer was purified with silica gel chromatography and afforded eight fractions (Fr. EA-Fr. EH) according to TLC plates. Fr. EB (26.8 g) was further chromatographed on CC (MCI, MeOH/H_2_O, from 30:70 to 95:5) to give nine fractions (Fr. EB1–EB9). Compounds **2** (MeOH/H_2_O, 56:44, t_R_ 27.0 min, 12.5 mg), **3** (MeCN/H_2_O, 45:55, t_R_ 37.0 min, 7.0 mg), **19** (MeOH/H_2_O, 40:60, t_R_ 48.0 min, 2.5 mg), and **20** (MeOH/H_2_O, 40:60, t_R_ 57.0 min, 4.2 mg) were purified from Fr. EB5 through semi-preparative HPLC.

Teucrifaride A (**1**): Yellow oil; UV (MeOH) λ_max_ (log *ε*): 200 nm: (2.87); ${[\alpha ]}_{\text{D}}^{25}$ +14.21 (0.02, MeOH); IR (KBr) *ν*_max_: 3444, 2935, 1786, 1678, 1118, 1010 cm^−1^; 1D NMR data: see Tables 1 and 2. HR-ESI-MS [M + Na]^+^: *m/z* 367.1149 (cacld for C_19_H_20_O_6_Na^+^, 367.1152).

Teucrifaride B (**2**): Yellow oil; UV (MeOH) λ_max_ (log *ε*): 200 (2.87) nm, 254 (2.00) nm; ${[\alpha ]}_{\text{D}}^{20}$ +34.62 (0.03, MeOH); IR (KBr) *ν*_max_: 2933, 2866, 1734, 1676, 1558, 1508, 1155 cm^−1^; 1D NMR NMR data: see Tables 1 and 2; HR-ESI-MS [M + Na]^+^: *m/z* 353.1346 (cacld for C_19_H_22_O_5_Na^+^, 353.1359).

Teucrifaride C (**3**): White and amorphous powder; UV (MeOH) λ_max_ (log *ε*): 206 nm: (3.28); ${[\alpha ]}_{\text{D}}^{20}$ −54.17 (0.02, MeOH); IR (KBr) *ν*_max_: 3525, 2939, 1689, 1180 cm^−1^; 1D NMR data: see Tables 1 and 2; HR-ESI-MS [M + Na]^+^: *m/z* 411.1408 (cacld for C_21_H_24_O_7_Na^+^, 411.1414).

Teucrifaride D (**4**): Colorless acicular crystal; mp 151–152 ℃; UV (MeOH) λ_max_ (log *ε*): 208 nm: (3.68); ${[\alpha ]}_{\text{D}}^{20}$+ 27.03 (0.01, MeOH); IR (KBr) *ν*_max_: 3155, 2970, 2939, 1774, 1751 cm^−1^; 1D NMR data: see Tables 1 and 2; HR-ESI–MS [M + Na]^+^: *m/z* 425.1559 (cacld for C_22_H_26_O_7_Na^+^, 425.1571).

### Single-crystal X-ray diffraction analysis of 4 and 10

A suitable crystal of compounds **4** and **10** were chosen and subjected to X-ray diffraction analysis on a XtaLAB AFC12 single X-ray diffractometer. The crystal was maintained at 100.00 (10) K for 4, and at 99.99 (10) K for 10, during data collection. The structural analysis was initiated using the Olex2 software, where the crystal structures were determined by the SHELXT program with intrinsic phasing. Subsequently, the structures were refined using the SHELXL refinement package, employing least squares minimization techniques [30]. The crystallographic data (CCDC.2383165 for 4 and CCDC.2383166 for 10) can be obtained free of charge via www.ccdc.cam.ac.uk/conts/retrieving.html.

### ECD calculations

ECD calculation methods were the same with previous reports [30].

### Cell viability assay

HT22 cells were grown in a culture medium consisting of DMEM (from HyCyte) supplemented with 1% penicillin/streptomycin (ECOTOP) and 10% fetal bovine serum (HyCyte). The culture was maintained in a 5% CO2 environment at a temperature of 37 ℃ within an incubator. HT22 cells were plated at a density of 3000 cells per well in 96-well plates and allowed to attach for 24 h. Subsequently, RSL3 (1 μM) or erastin (1 μM) along with different concentrations of compounds were introduced. After 24 h, MTT reagent (10 μL, 5 mg/mL) was introduced to each well and incubated for an additional 4 h. Following this, the formazan crystals were solubilized using 150 μL of DMSO. The plates were softly shaken for 15 min at ambient temperature, and then tested the absorbance at 490 nm using a microplate reader.

### Intracellular reactive oxygen species detection

HT22 cells were cultured at 300,000 cells/well in 6-well plates for 24 h. RSL3 (1 μM) and three different concentrations (5, 10, 20 μM) of compound **1** and Fer-1 (1 μM) were added to plates. After a 4 h reaction, the supernatant medium was aspirated off and H_2_PCFDA (1 mL, 10 μM) was added in an incubator and incubated for 40 min (37 ℃), then washed twice with serum-free medium and photographed with a fluorescence microscope under dark conditions.

## Supplementary Information

Additional file 1.

## Data Availability

The data that support the findings of this study are openly available in the Science Data Bank at https://doi.org/https://doi.org/

## References

[1] Flora of China Editorial Committee of Chinese Academy of Sciences. Flora of China. Beijing: Science Press; 1990.

[2] Zhang K, Wang XL, Li YK, Li J, Xv KP, Tan GS. Chemical constituents and pharmacological activities of genus *Teucrium*. Central South Pharm. 2016;14(7):735–41.

[3] Aydogan F, Boga M, Khan SI, Zulfiqar F, Khan IA, Ali Z. Phytochemical investigation of *Teucrium pruinosum* and biological potential assessment of the isolated diterpenoids. Biochem Syst Ecol. 2022;105: 104545.

[4] Kurimoto S, Wakabayashi K, Sasaki YF, Nakamura T, Kubota T. Teuchamaedol A, a new neo-clerodane diterpenoid from the aerial parts of *Teucrium chamaedrys*. Tetrahedron Lett. 2022;100: 153890.

[5] Lv HW, Luo JG, Zhu MD, Zhao HJ, Kong LY. *neo*-Clerodane diterpenoids from the aerial parts of *Teucrium fruticans* cultivated in China. Phytochemistry. 2015;119:26–31. 26454794 10.1016/j.phytochem.2015.09.011

[6] Tan Q, Fang Y, Peng X, Zhou H, Xu J, Gu Q. A new ferroptosis inhibitor, isolated from *Ajuga nipponensis*, protects neuronal cells via activating NRF2-antioxidant response elements (AREs) pathway. Bioorg Chem. 2021;115: 105177. 34303035 10.1016/j.bioorg.2021.105177

[7] Li S, Xu D, Jia J, Zou W, Liu J, Wang Y, Zhang K, Zheng X, Ma YY, Zhang X, Zhao DG. Structure and anti-inflammatory activity of *neo*-clerodane diterpenoids from *Scutellaria barbata*. Phytochemistry. 2023;213: 113771. 37352949 10.1016/j.phytochem.2023.113771

[8] Peng X, Tan Q, Zhang Z, Wu D, Xu J, Zhou H, Gu Q. Discovery of *neo*-clerodane diterpenoids from *Ajuga campylantha* as neuroprotective agents against ferroptosis and neuroinflammation. J Nat Prod. 2023;86(8):2006–21. 37566645 10.1021/acs.jnatprod.3c00447

[9] Krishna Kumari GN, Aravind S, Balachandran J, Ganesh MR, Soundarya Devi S, Rajan SS, Malathi R, Ravikumar K. Antifeedant neo-clerodanes from Teucrium tomentosum Heyne (Labiatae). Phytochemistry. 2003. 10.1016/S0031-9422(03)00510-7. 14568078 10.1016/s0031-9422(03)00510-7

[10] Kheawchaum S, Mahidol C, Thongnest S, Boonsombat J, Batsomboon P, Sitthimonchai S, Ruchirawat S, Prawat H. Ent-abietane diterpenoid lactone glycosides and a phenolic glycoside from Phlogacanthus pulcherrimus T Anderson with cytotoxic and cancer chemopreventive activities. Phytochemistry. 2022;201:113261. 35662549 10.1016/j.phytochem.2022.113261

[11] Hunan Academy of Chinese Medicine. Pharmaceutical Records of Hunan. Changsha: Hunan People’s Publishing House; 1979.

[12] National Institutes for Food and Drug Contro. Yunnan Institute for Drug Control. In: Annals of Chinese national medicine. Beijing: People’s Medical Publishing House; 1984.

[13] Xie N, Ming ZD, Zhao SX, Feng R. Flavones from *Teurium quadrifarium*. J China Pharm Univ. 1991;22(4):200–2.

[14] Zhu YY, Li GY. Studies on the diterpenoids of *Teucrium quadrifarium* Buch-Ham. Acta Pharm Sin B. 1993;28(9):679–83. 8010014

[15] Aydoğan F, Ali Z, Zulfiqar F, Karaalp C, Khan IA, Bedir E. neo-clerodanes from Teucrium divaricatum subsp. divaricatum and their biological activity assessment. Phytochem Lett. 2023;54:45–9.

[16] Simoes F, Rodríguez B, Bruno M, Piozzi F, Savona G, Arnold NA. *neo*-Clerodane diterpenoids from *Teucrium kotschyanum*. Phytochemistry. 1989;28(10):2763–8.

[17] Lv HW, Luo JG, Meng DZ, Shan SM, Kong LY. Teucvisins A-E, five new *neo*-clerodane diterpenes from *Teucrium viscidum*. Chem Pharm Bull. 2014;62(5):472–6. 10.1248/cpb.c13-00990 24789929

[18] Aoyagi Y, Yamazaki A, Nakatsugawa C, Fukaya H, Takeya K, Kawauchi S, Izumi H. Salvileucalin B, a novel diterpenoid with an unprecedented rearranged neoclerodane skeleton from *Salvia leucantha* Cav. Org Lett. 2008;10(20):4429–32. 18788744 10.1021/ol801620u

[19] Piozzi F, Savona G, Paternostro M, Rodriguez BP. New clerodane diterpenoids from *Teucrium spinosum* L. Heterocycles. 1980;14:193.

[20] Bruno M, Piozzi F, Savona G, De La Torre MC, Rodríguez B. *neo*-Clerodane diterpenoids from *Teucrium canadense*. Phytochemistry. 1989;28(12):3539–41.

[21] Li W, Wang RM, Pan YH, Zhao YY, Yuan FY, Huang D, Tang GH, Bi HC, Yin S. Crotonpenoids A and B, two highly modified clerodane diterpenoids with a tricyclo[7.2.1.02,7]dodecane core from Croton yanhuii: isolation, structural elucidation, and biomimetic semisynthesis. Org Lett. 2020;22(11):4435–9. 32452687 10.1021/acs.orglett.0c01443

[22] Xu G, Peng L, Niu X, Zhao Q, Li R, Sun H. Novel diterpenoids from *Salvia dugesii*. Helv Chim Acta. 2004;87(4):949–55.

[23] Jiang YJ, Su J, Shi X, Wu XD, Chen XQ, He J, Shao LD, Li XN, Peng LY, Li RT, Zhao QS. *neo*-Clerodanes from the aerial parts of *Salvia leucantha*. Tetrahedron. 2016;72(35):5507–14.

[24] Papanov GY, Malakov PY. New furanoid diterpenes from T*eucrium scordium* L. Zeitschrift für Naturforschung B. 1981;36(1):112–3.

[25] Ye D, Shu LP, Qiang Z, Xun L, Li SD. Clerodane diterpenoids from *Kinostemon alborubrum*. Helv Chim Acta. 2002;85(8):2547–52.

[26] Maria C, Rodríguez B, Bruno M, Savona G, Piozzi F, Servettaz O. *neo*-clerodane diterpenoids from *Teucrium micropodioldes*. Phytochemistry. 1988;27(1):213–6.

[27] Gacs-Baitz E, Kajtar M, Papanov G, Malakov P. Carbon-13 NMR spectra of some furanoid diterpenes from *Teucrium species*. Heterocycles. 1982;19:539–50.

[28] Mbwambo ZH, Foubert K, Chacha M, Kapingu MC, Magadula JJ, Moshi MM, Lemiere F, Goubitz K, Fraanje J, Peschar R, Vlietinck A, Apers S, Pieters L. New furanoditerpenoids from *Croton jatrophoides*. Planta Med. 2009;75(3):262–7. 19090454 10.1055/s-0028-1088383

[29] Shi HW, Jiang XF, Cao LD, Peng X, Tan QY, Teng XF, Gu Q, He L. Chemical constituents of *Ajuga forrestii* and their anti-ferroptosis activity. Fitoterapia. 2023;166: 105461. 36804655 10.1016/j.fitote.2023.105461

[30] Zhao X, Zheng ZP, Chen C, Wang H, Liu HF, Li JY, Sun C, Lou HY, Pan WD. New clerodane diterpenoids from *Callicarpa pseudorubella* and their antitumor proliferative activity. Fitoterapia. 2024;174: 105878. 38417683 10.1016/j.fitote.2024.105878

