# Supplementary material for: Novel neo-clerodane diterpenoids from Teucrium quadrifarium and their anti-ferroptosis effect
Source: Nat Prod Bioprospect. 2025 Jan 6;15(1):8. doi: 10.1007/s13659-024-00489-1 (PMC11700957; doi:10.1007/s13659-024-00489-1)
Supplement: Supplementary file 1 — Additional file 1. [file 13659_2024_489_MOESM1_ESM.docx]

**Novel** ***neo*-clerodane diterpenoids from *Teucrium* *quadrifarium* and their anti-ferroptosis effect**

Huan Wang,^a,b#^ Han-Fei Liu,^a,b#^ Yu-Qiong Liao,^a,b^ Fen-Cong Pan,^a,b^ Jin-Yu Li,^a,b*^ Hua-Yong Lou ^a,b*^, Wei-Dong Pan^a*^

^a^ *Guizhou Medical University, State Key Laboratory of Functions and Applications of Medicinal Plants, Guiyang 550014, China*

^b^ *Natural Products Research Center of Guizhou Province, Guiyang 550014, China.*

*^#^These authors contributed equally to this work.*

Corresponding author information: [wdpan@163.com](mailto:wdpan@163.com); [lijinyu20080@126.com](mailto:lijinyu20080@126.com); [loouhy@126.com](mailto:loouhy@126.com)

**Table of content**

[Spectra of physico-chemical properties of 1 4](#_Toc175136376)

[Figure S1. ^1^H NMR spectrum of 1. 4](#_Toc175136377)

[Figure S2. ^13^C NMR spectrum of 1. 4](#_Toc175136378)

[Figure S3. DEPT spectrum of 1. 5](#_Toc175136379)

[Figure S4. ^1^H–^1^H COSY spectrum of 1. 5](#_Toc175136380)

[Figure S5. HMQC spectrum of 1. 6](#_Toc175136381)

[Figure S6. HMBC spectrum of 1. 6](#_Toc175136382)

[Figure S7. NOESY spectrum of 1. 7](#_Toc175136383)

[Figure S8. HR-ESI-MS spectrum of 1. 7](#_Toc175136384)

[Figure S9. UV spectrum of 1. 8](#_Toc175136385)

[Figure S10. IR spectrum of 1. 8](#_Toc175136386)

[Figure S11. CD spectrum of 1. 9](#_Toc175136387)

[Spectra of physico-chemical properties of 2 10](#_Toc175136388)

[Figure S12. ^1^H NMR spectrum of 2. 10](#_Toc175136389)

[Figure S13. ^13^C NMR spectrum of 2. 10](#_Toc175136390)

[Figure S14. DEPT spectrum of 2. 11](#_Toc175136391)

[Figure S15. ^1^H–^1^H COSY spectrum of 2. 11](#_Toc175136392)

[Figure S16. HMQC spectrum of 2. 12](#_Toc175136393)

[Figure S17. HMBC spectrum of 2. 12](#_Toc175136394)

[Figure S18. NOESY spectrum of 2. 13](#_Toc175136395)

[Figure S19. HR-ESI-MS spectrum of 2. 13](#_Toc175136396)

[Figure S20. UV spectrum of 2. 14](#_Toc175136397)

[Figure S21. IR spectrum of 2. 14](#_Toc175136398)

[Figure S22. CD spectrum of 2. 15](#_Toc175136399)

[Spectra of physico-chemical properties of 3 16](#_Toc175136400)

[Figure S23. ^1^H NMR spectrum of 3. 16](#_Toc175136401)

[Figure S24. ^13^C NMR spectrum of 3. 16](#_Toc175136402)

[Figure S25. DEPT spectrum of 3. 17](#_Toc175136403)

[Figure S26. ^1^H–^1^H COSY spectrum of 3. 17](#_Toc175136404)

[Figure S27. HMQC spectrum of 3. 18](#_Toc175136405)

[Figure S28. HMBC spectrum of 3. 18](#_Toc175136406)

[Figure S29. NOESY spectrum of 3. 19](#_Toc175136407)

[Figure S30. HR-ESI-MS spectrum of 3. 19](#_Toc175136408)

[Figure S31. UV spectrum of 3. 20](#_Toc175136409)

[Figure S32. CD spectrum of 3. 20](#_Toc175136410)

[Spectra of physico-chemical properties of 4 21](#_Toc175136411)

[Figure S33. ^1^H NMR spectrum of 4. 21](#_Toc175136412)

[Figure S34. ^13^C NMR spectrum of 4. 21](#_Toc175136413)

[Figure S35. 1H–1H COSY spectrum of 4. 22](#_Toc175136414)

[Figure S36. HMQC spectrum of 4. 22](#_Toc175136415)

[Figure S37. HMBC spectrum of 4. 23](#_Toc175136416)

[Figure S38. NOESY spectrum of 4. 23](#_Toc175136417)

[Figure S39. HR-ESI-MS spectrum of 4. 24](#_Toc175136418)

[Figure S40. UV spectrum of 4. 24](#_Toc175136419)

[Table S1 b3lyp/6-311+g(d,2p) optimized lowest energy 3D conformer of compound 1 25](#_Toc175136420)

[Table S2 b3lyp/6-311+g(d,2p) optimized lowest energy 3D conformer of compound 2 26](#_Toc175136421)

[Table S3 b3lyp/6-311+g(d,2p) optimized lowest energy 3D conformer of compound 3 28](#_Toc175136422)

[Table S4 Crystal data and structure refinement for 4. 29](#_Toc175136423)

[Table S5  Crystal data and structure refinement for 10. 30](#_Toc175136424)

# Spectra of physico-chemical properties of 1

## Figure S1. ^1^H NMR spectrum of 1.


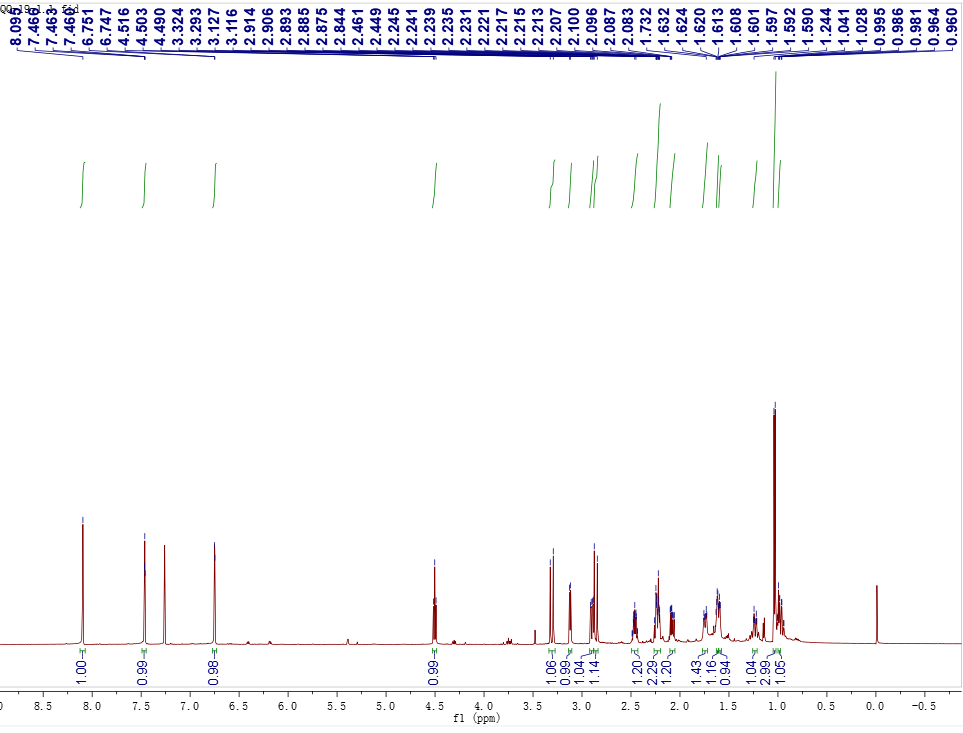


## Figure S2. ^13^C NMR spectrum of 1.


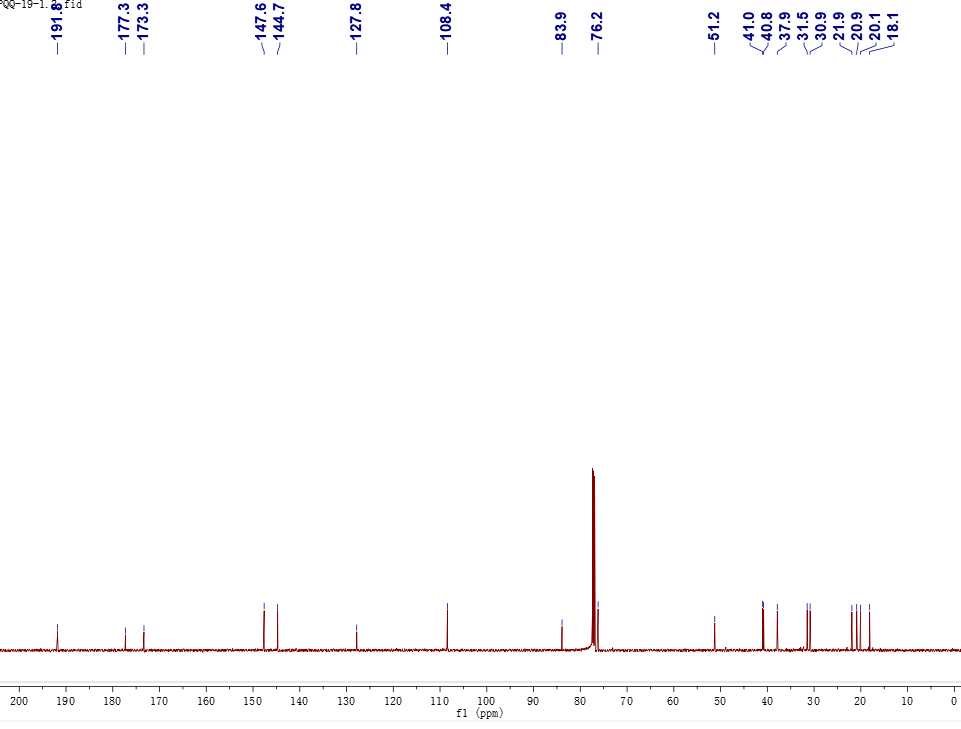


## Figure S3. DEPT spectrum of 1.


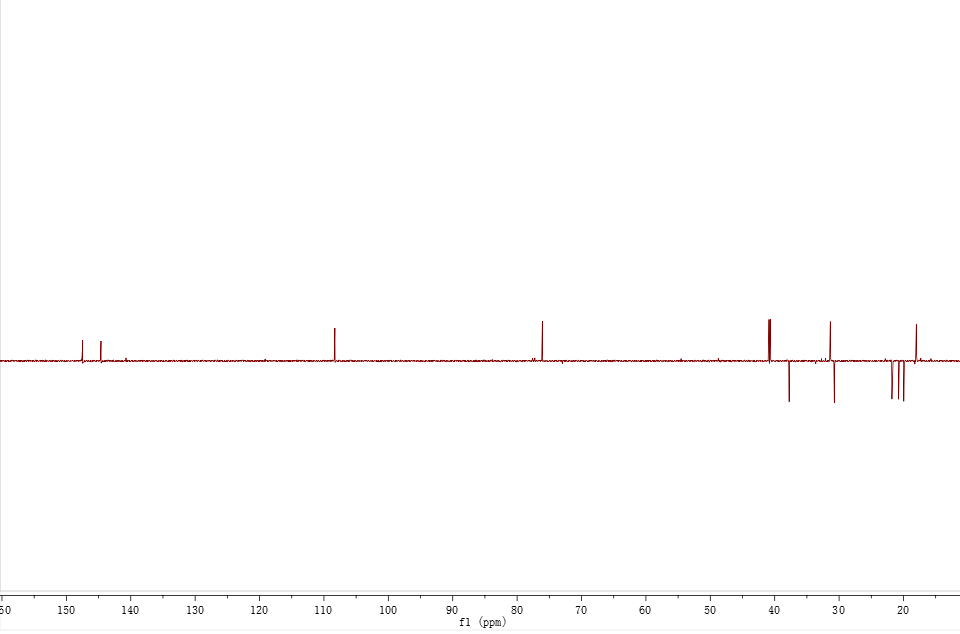


##

## Figure S4. ^1^H–^1^H COSY spectrum of 1.


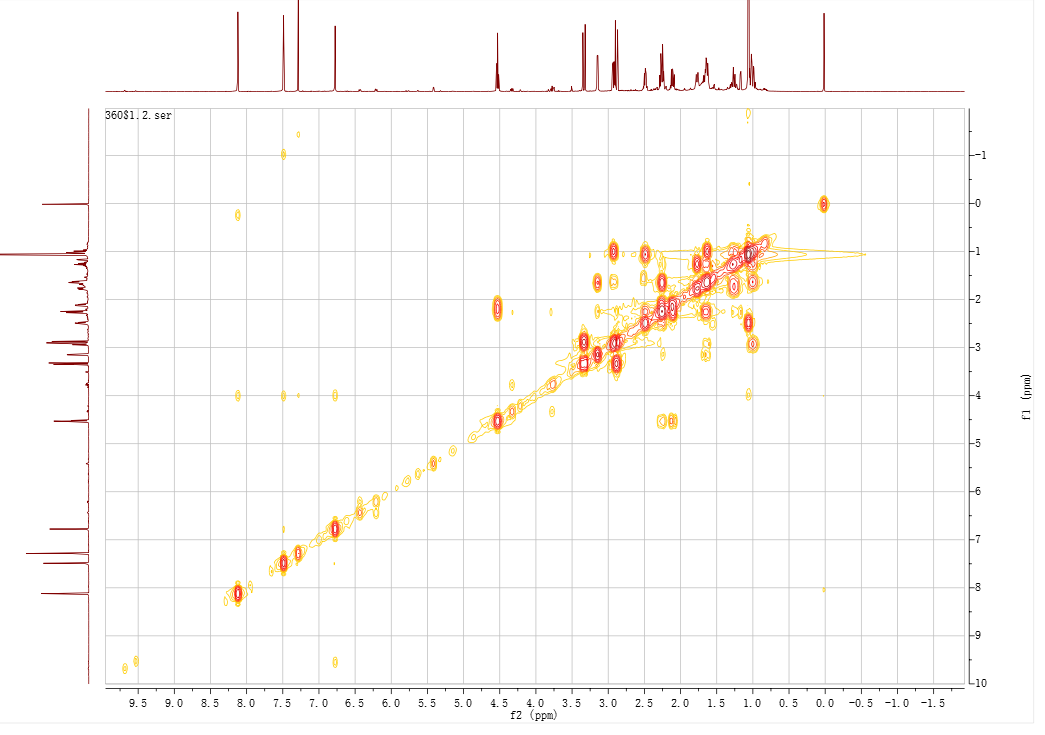


## Figure S5. HMQC spectrum of 1.


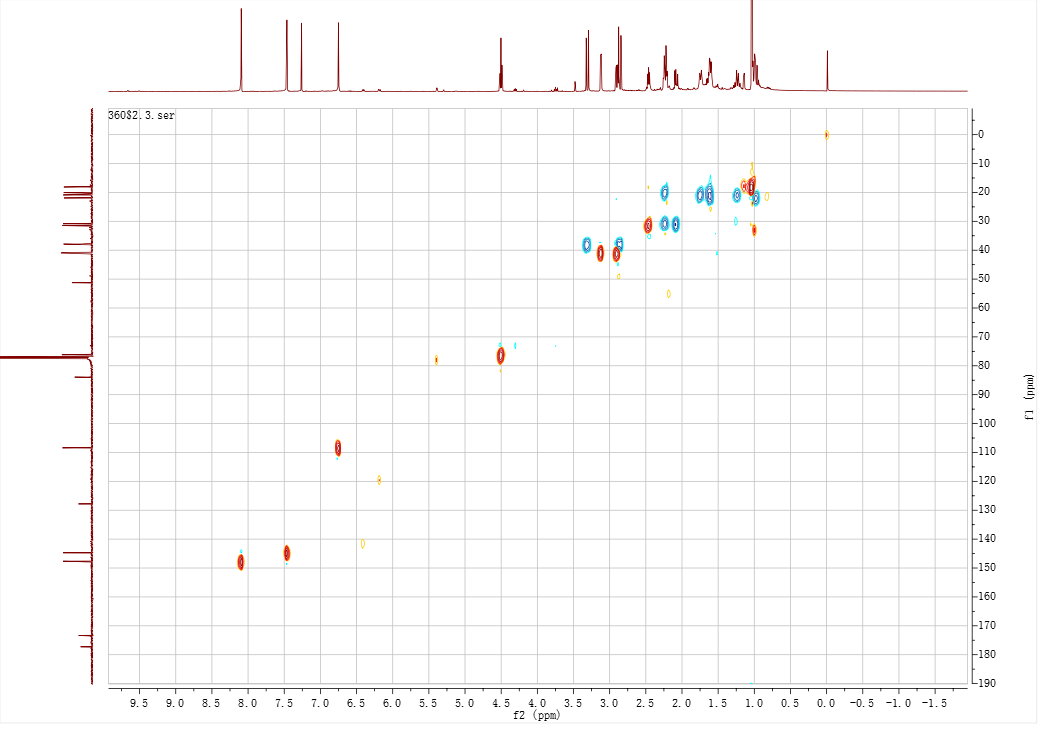


## Figure S6. HMBC spectrum of 1.


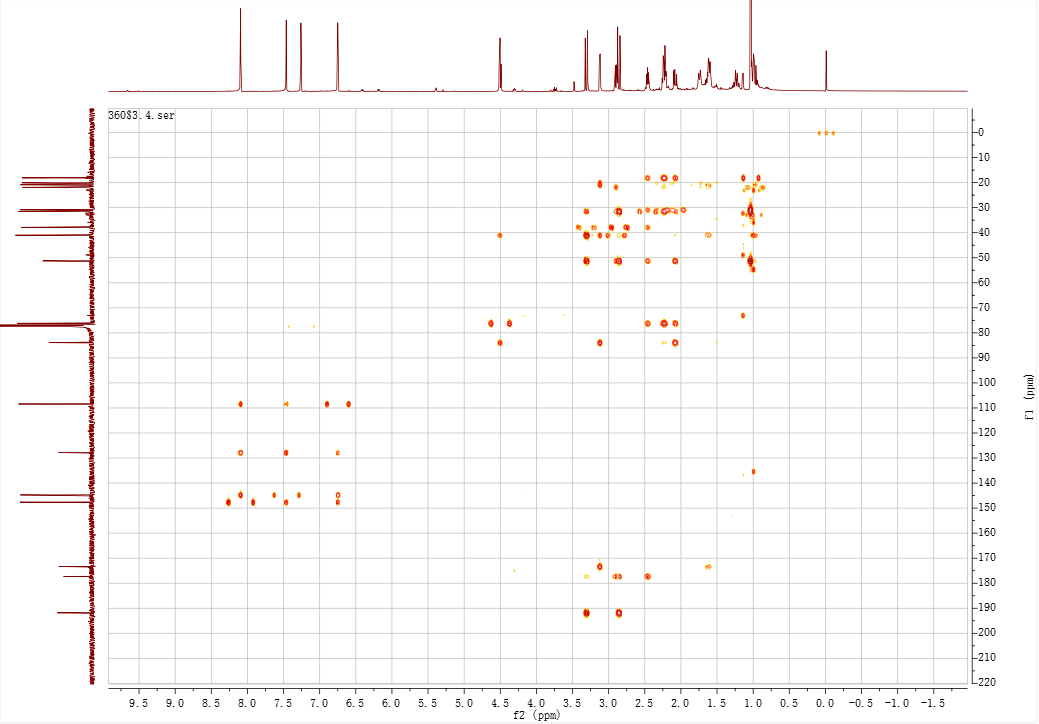


## Figure S7. NOESY spectrum of 1.


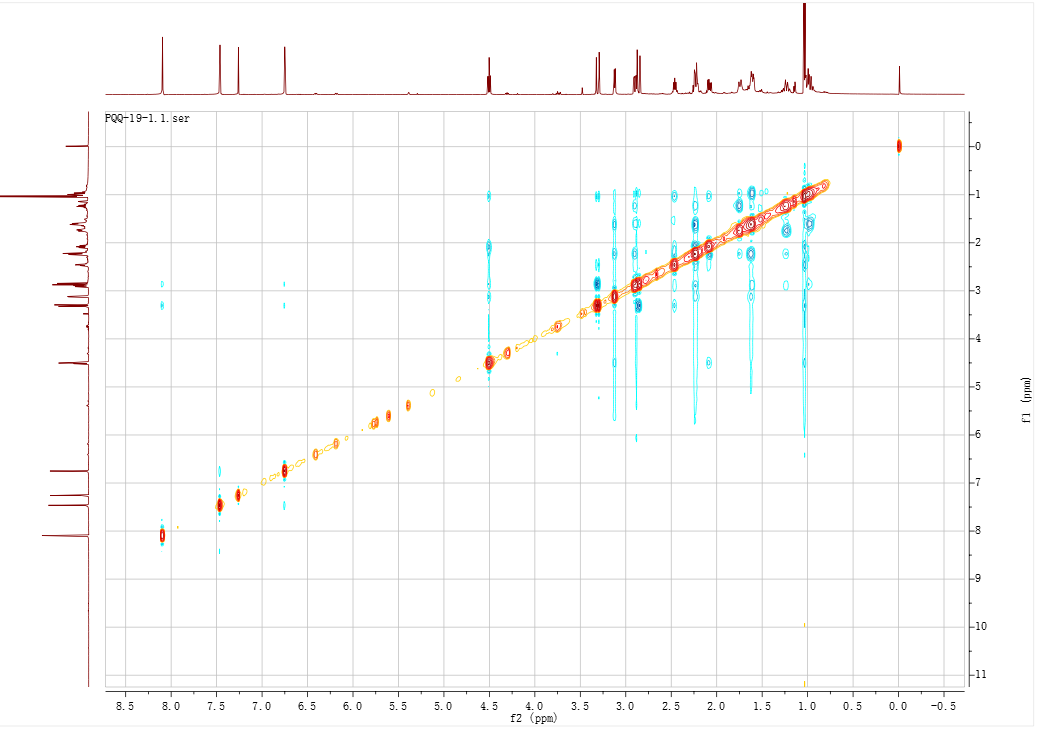


## Figure S8. HR-ESI-MS spectrum of 1.

## Figure S9. UV spectrum of 1.


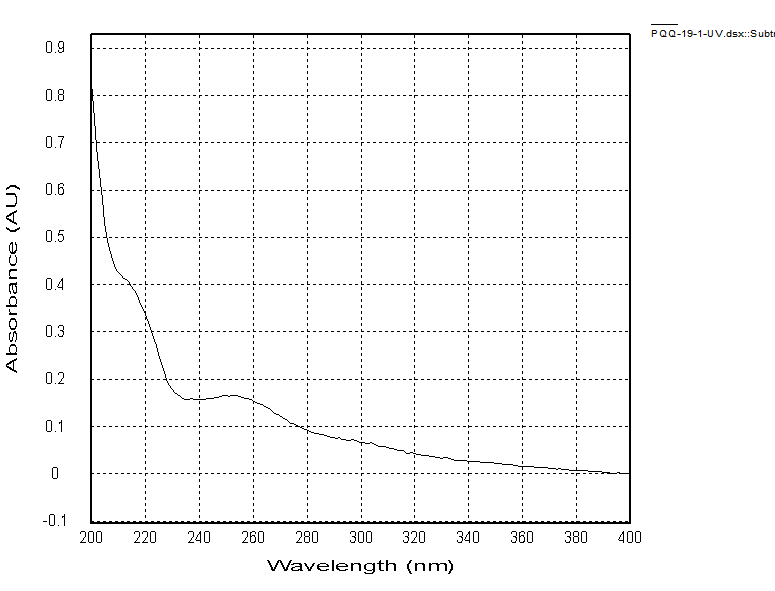


## Figure S10. IR spectrum of 1.


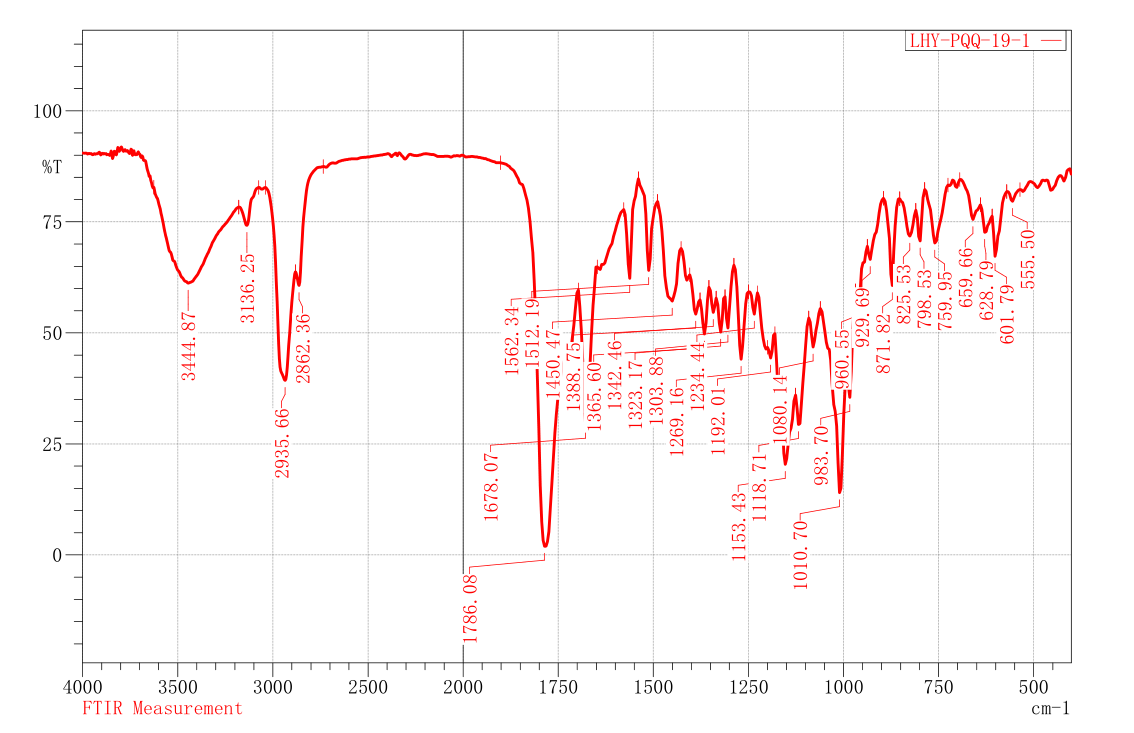


## Figure S11. CD spectrum of 1.


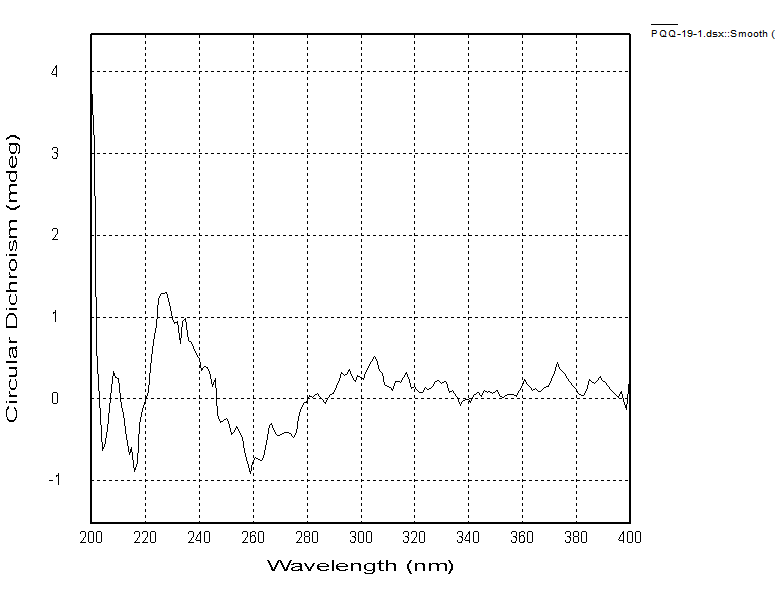


# Spectra of physico-chemical properties of 2

## Figure S12. ^1^H NMR spectrum of 2.


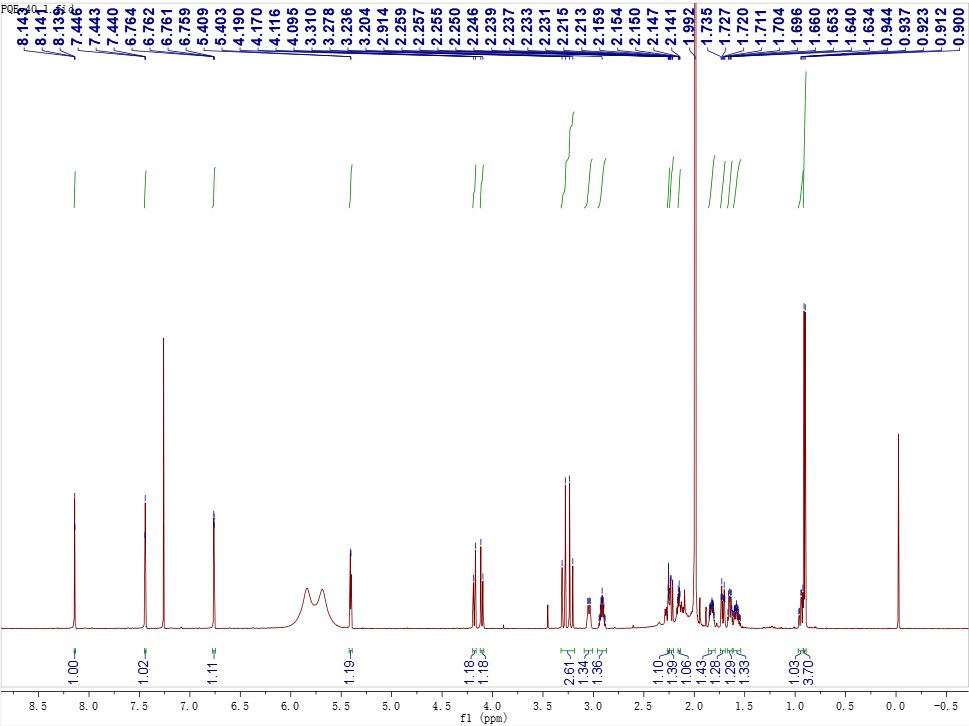


## Figure S13. ^13^C NMR spectrum of 2.


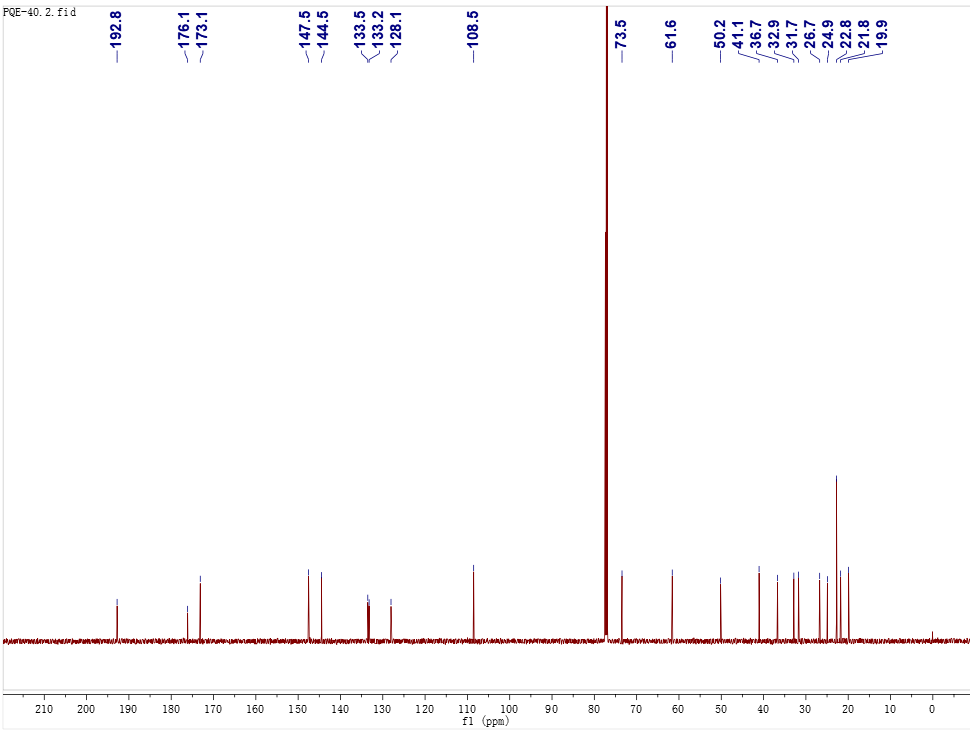


Figure S14. DEPT spectrum of 2.
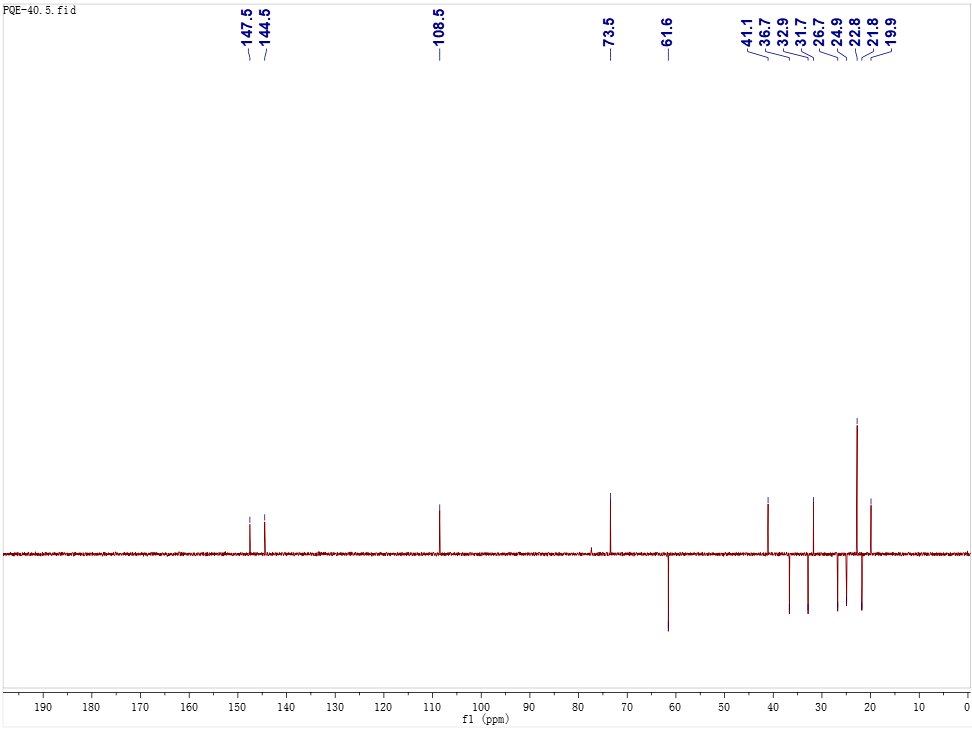


## Figure S15. ^1^H–^1^H COSY spectrum of 2.


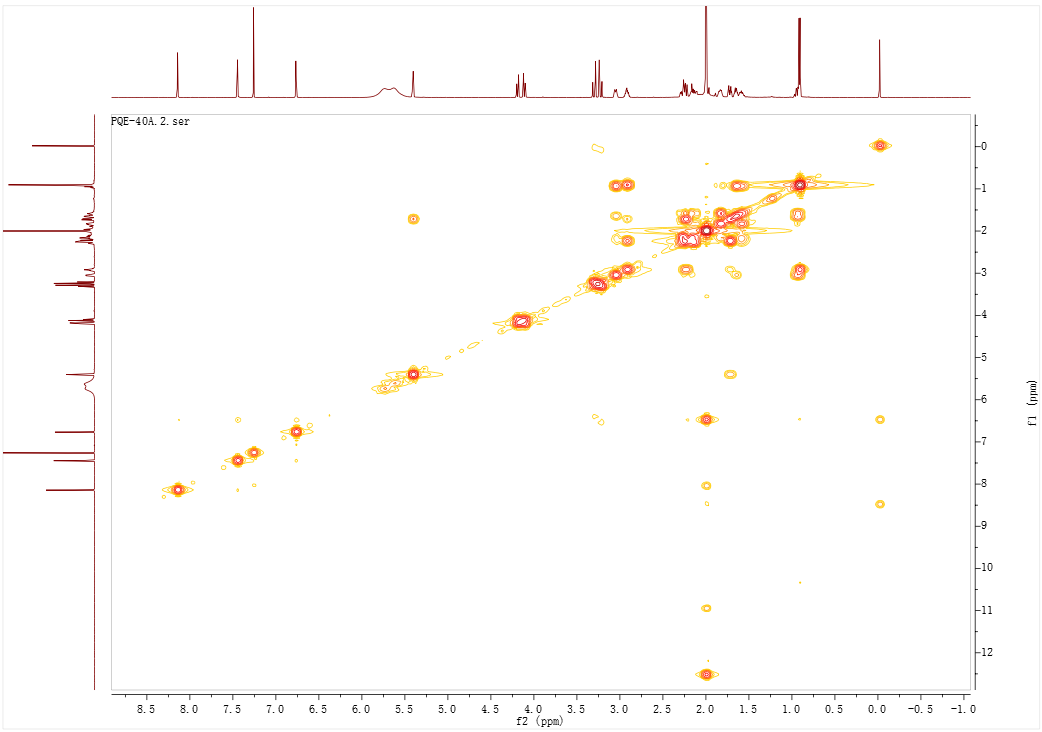


## Figure S16. HMQC spectrum of 2.


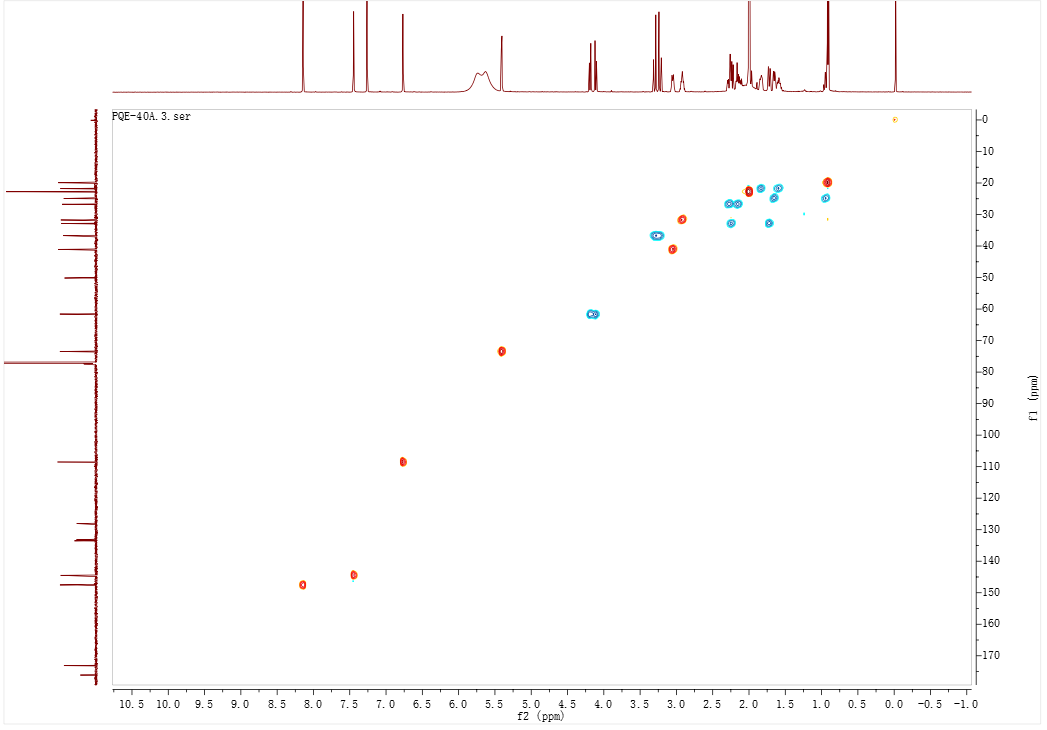


## Figure S17. HMBC spectrum of 2.


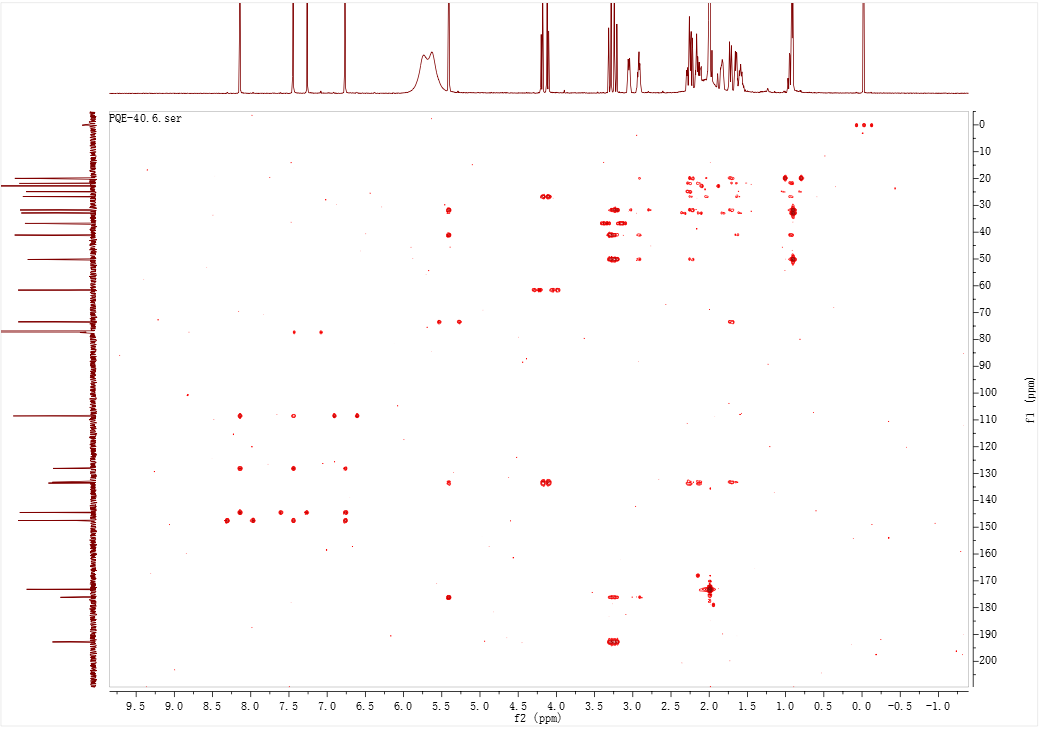


## Figure S18. NOESY spectrum of 2.


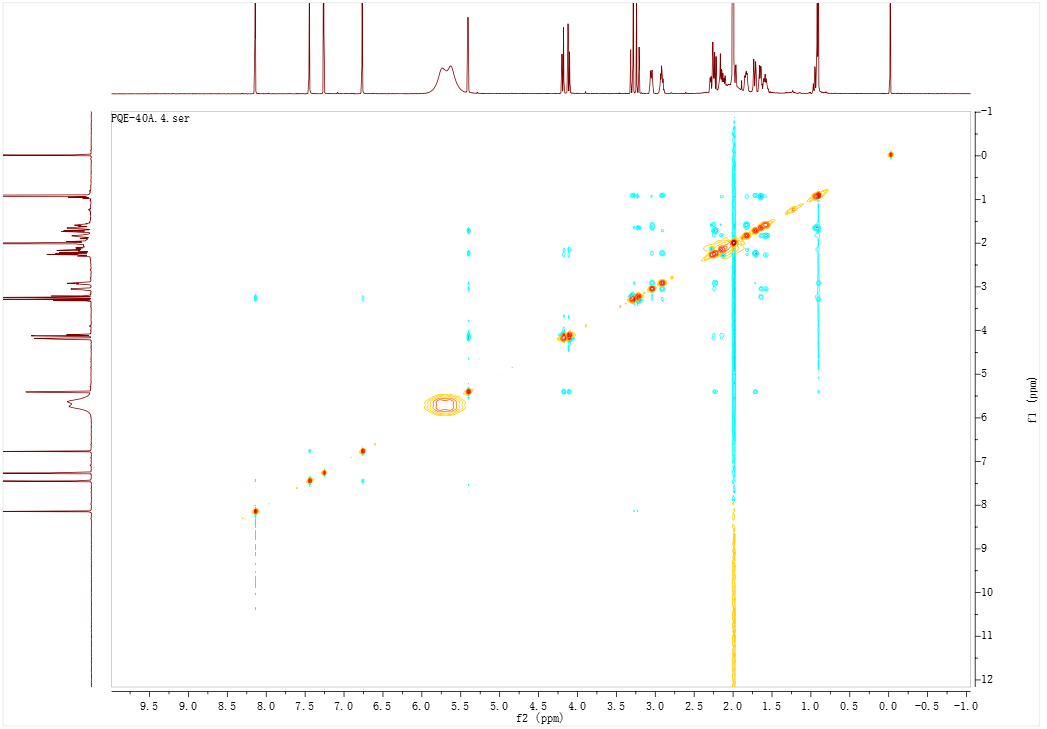


## Figure S19. HR-ESI-MS spectrum of 2.


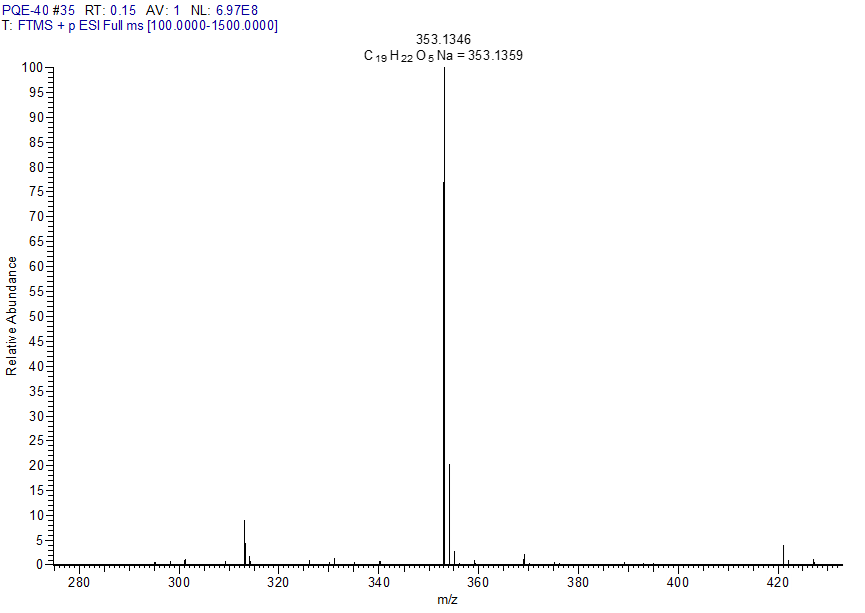


## Figure S20. UV spectrum of 2.


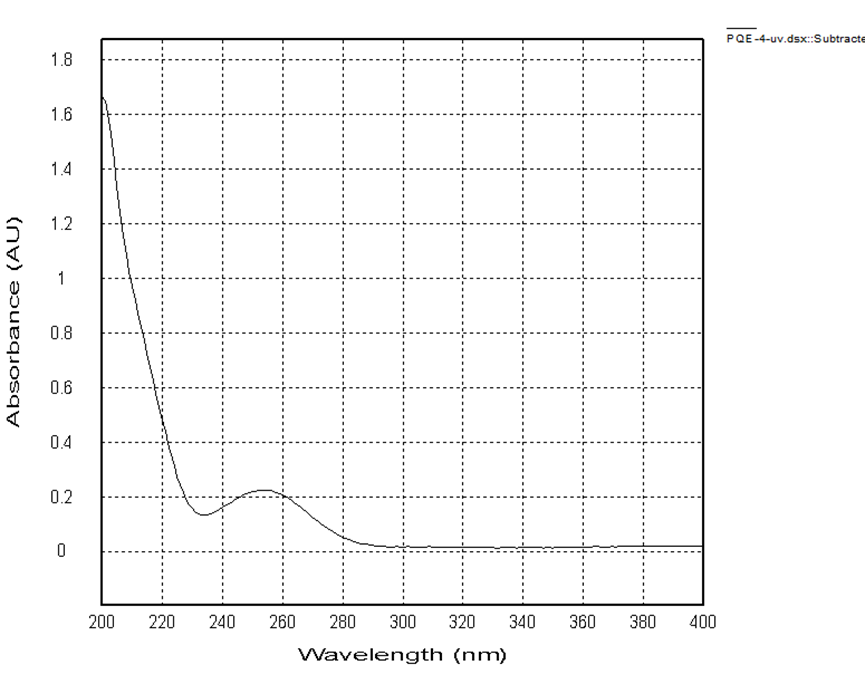


## Figure S21. IR spectrum of 2.


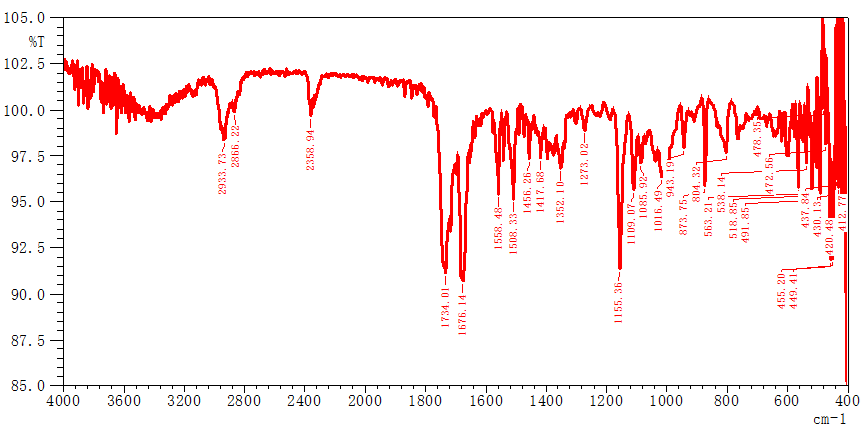


## Figure S22. CD spectrum of 2.


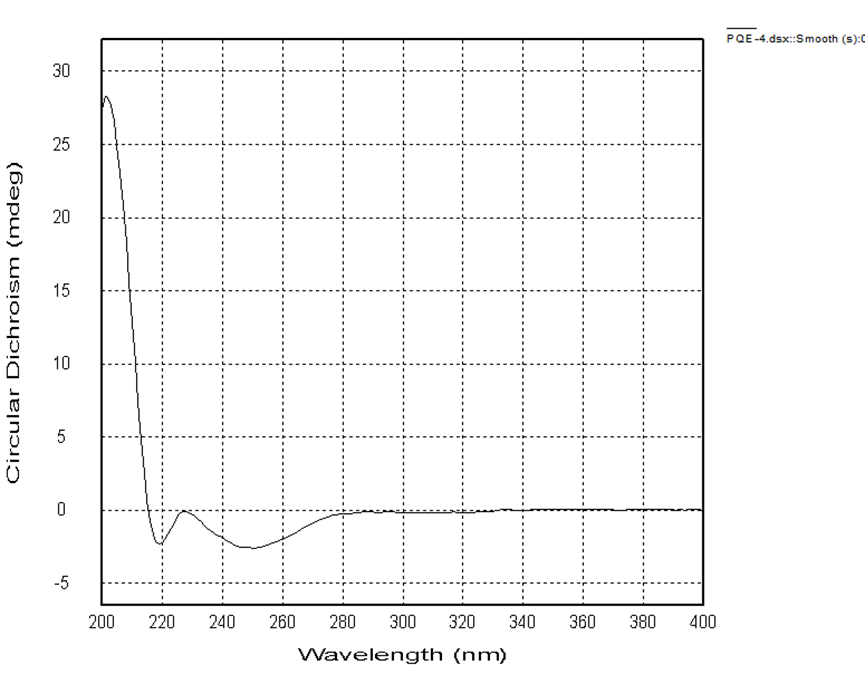


# Spectra of physico-chemical properties of 3

## Figure S23. ^1^H NMR spectrum of 3.


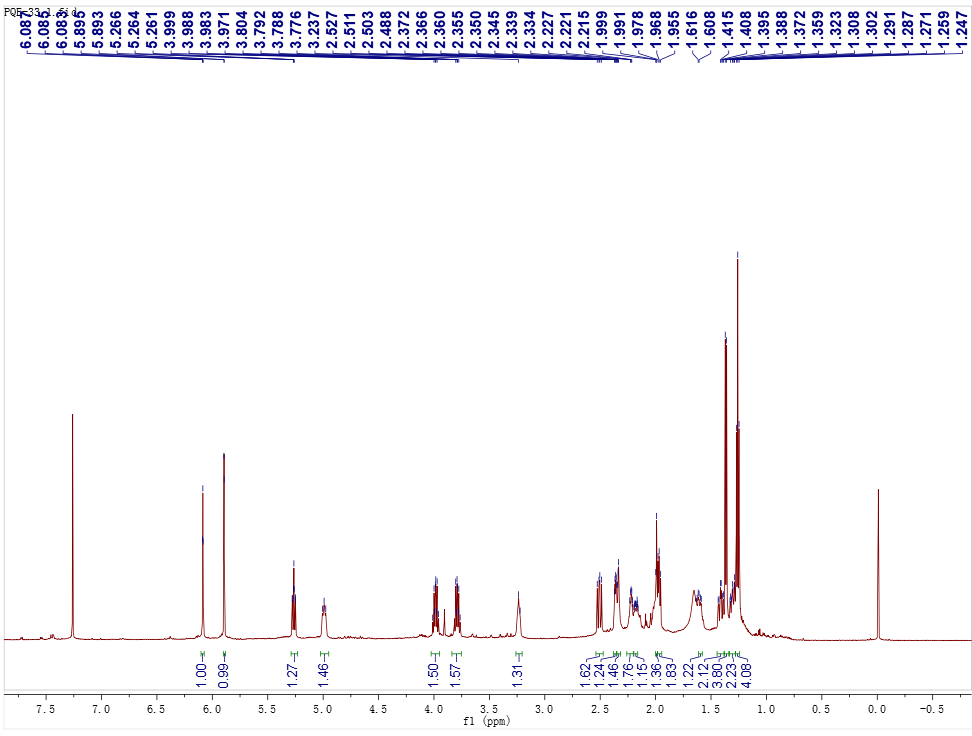


## Figure S24. ^13^C NMR spectrum of 3.


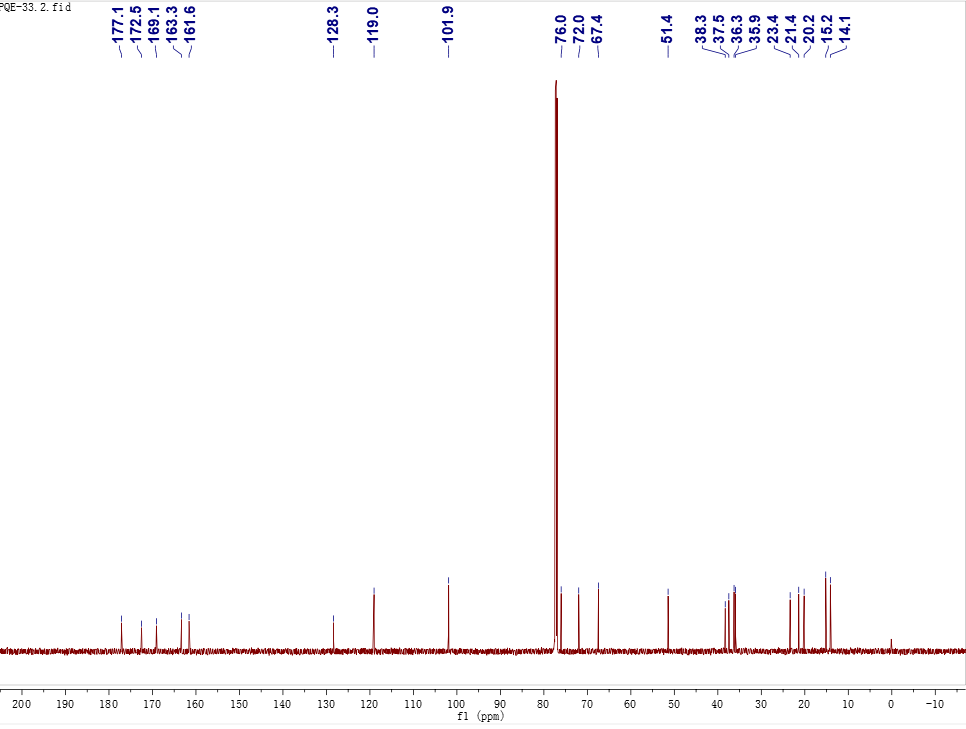


## Figure S25. DEPT spectrum of 3.


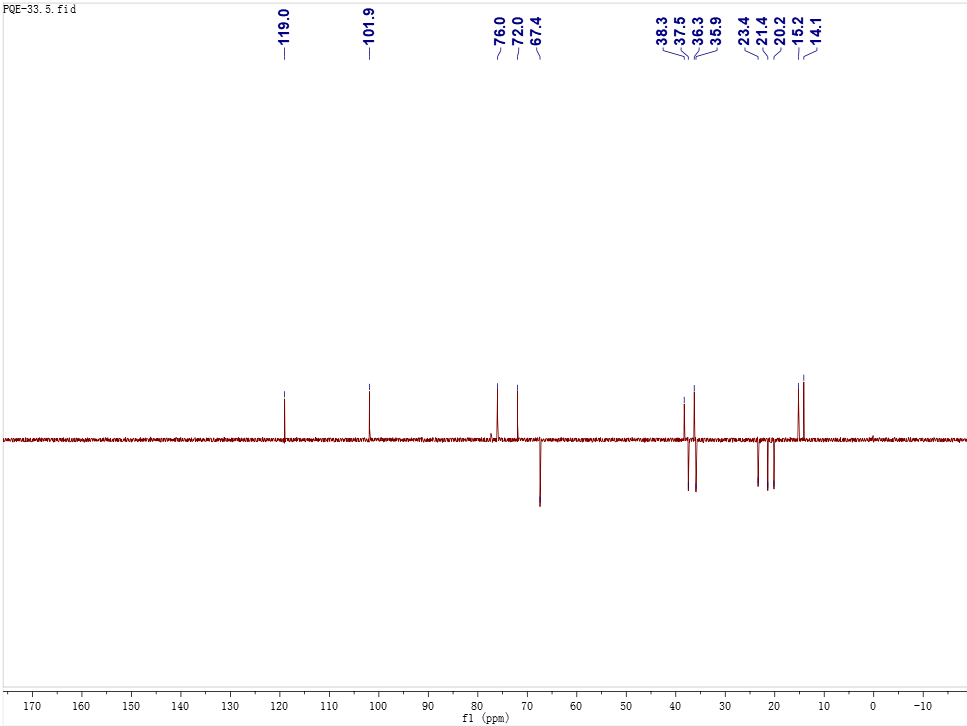


## Figure S26. ^1^H–^1^H COSY spectrum of 3.


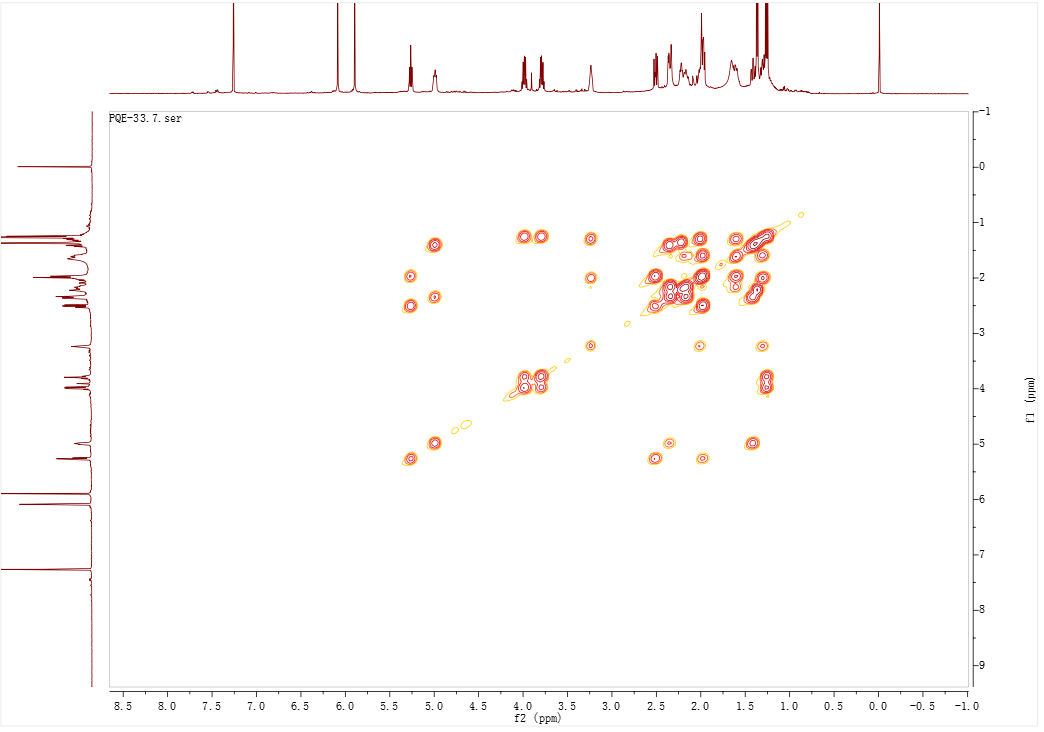


## Figure S27. HMQC spectrum of 3.


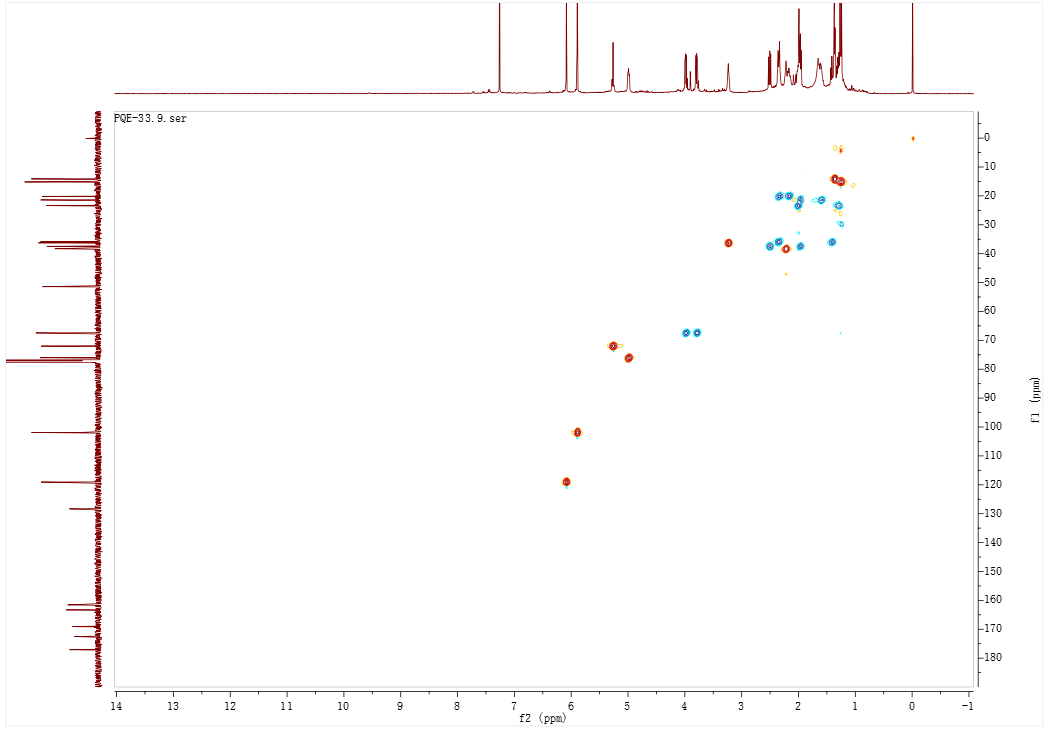


## Figure S28. HMBC spectrum of 3.


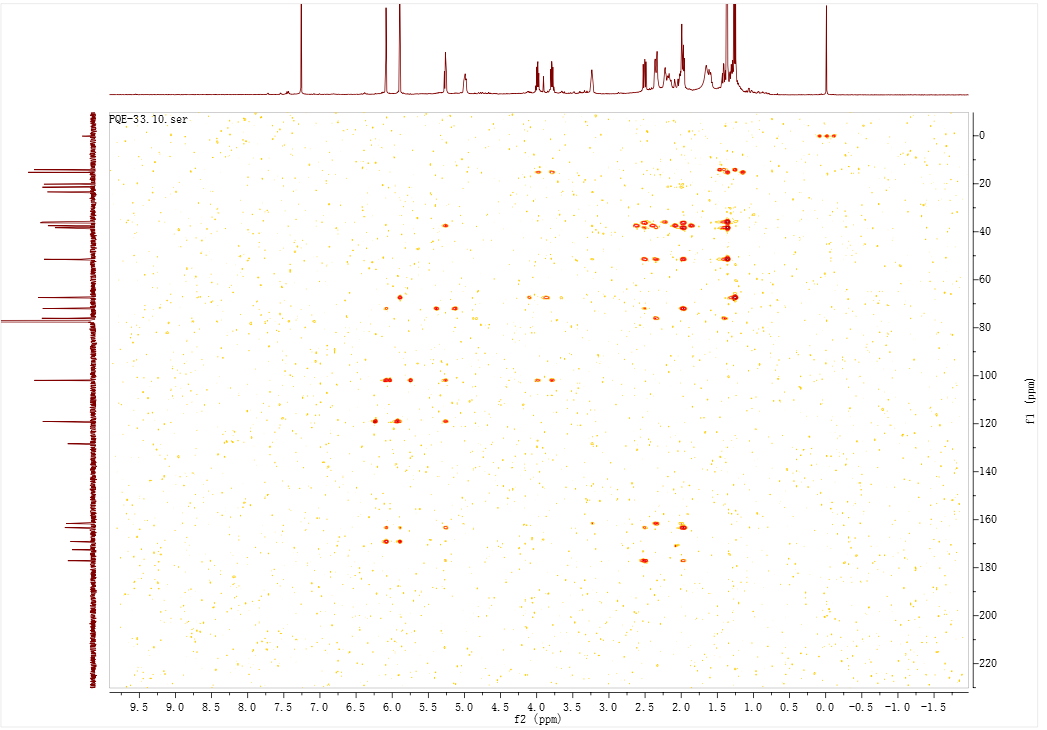


## Figure S29. NOESY spectrum of 3.


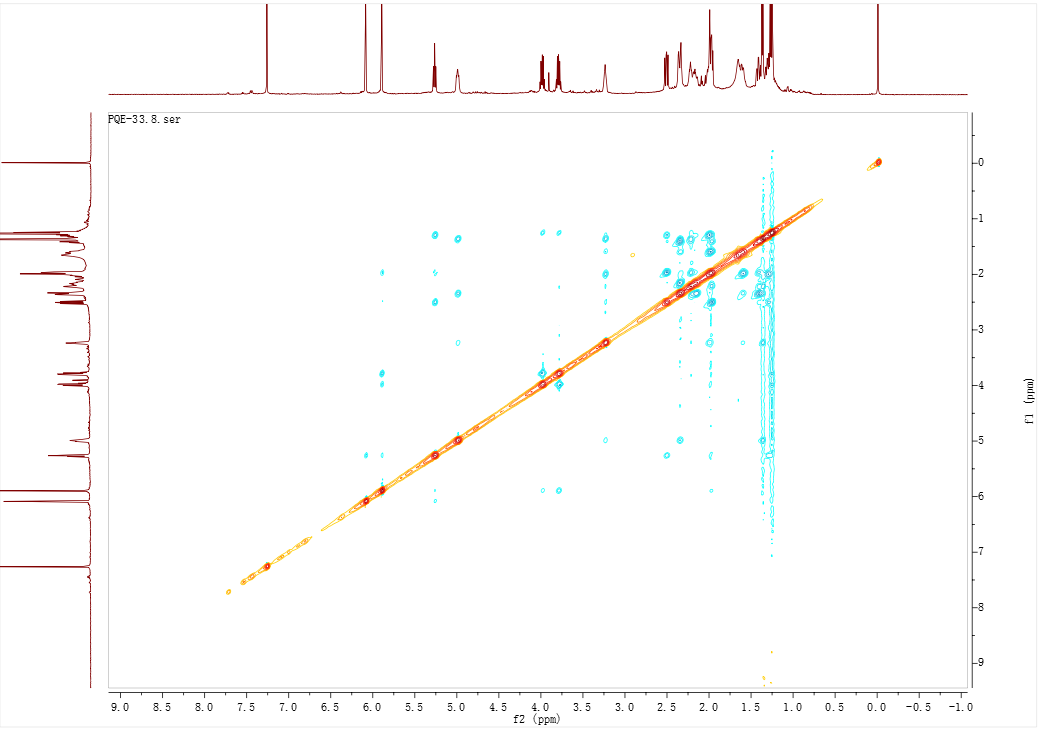


## Figure S30. HR-ESI-MS spectrum of 3.


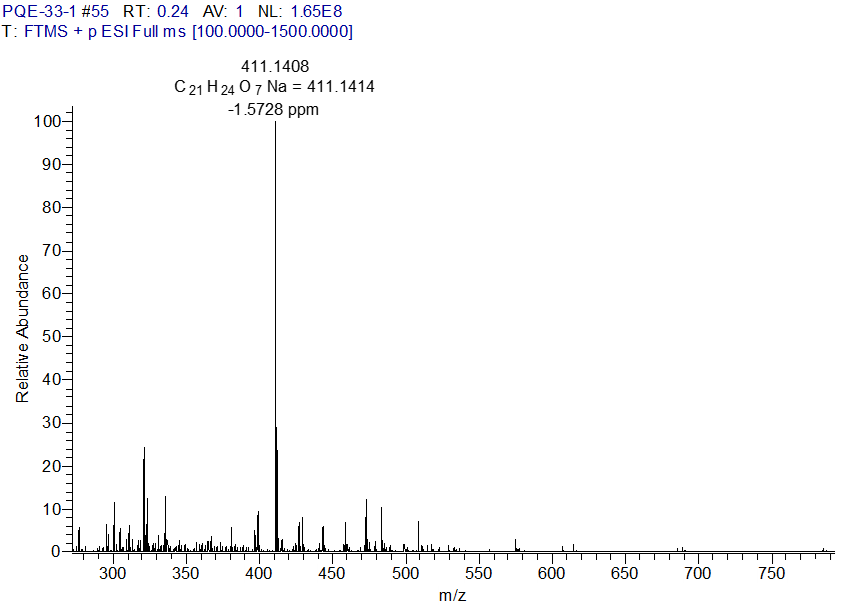


## Figure S31. UV spectrum of 3.


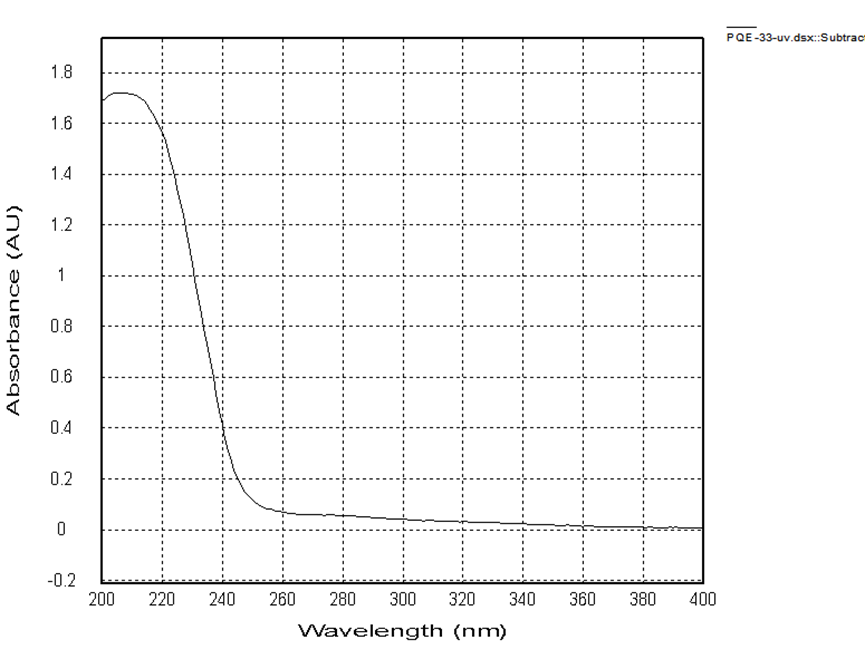


## Figure S32. CD spectrum of 3.


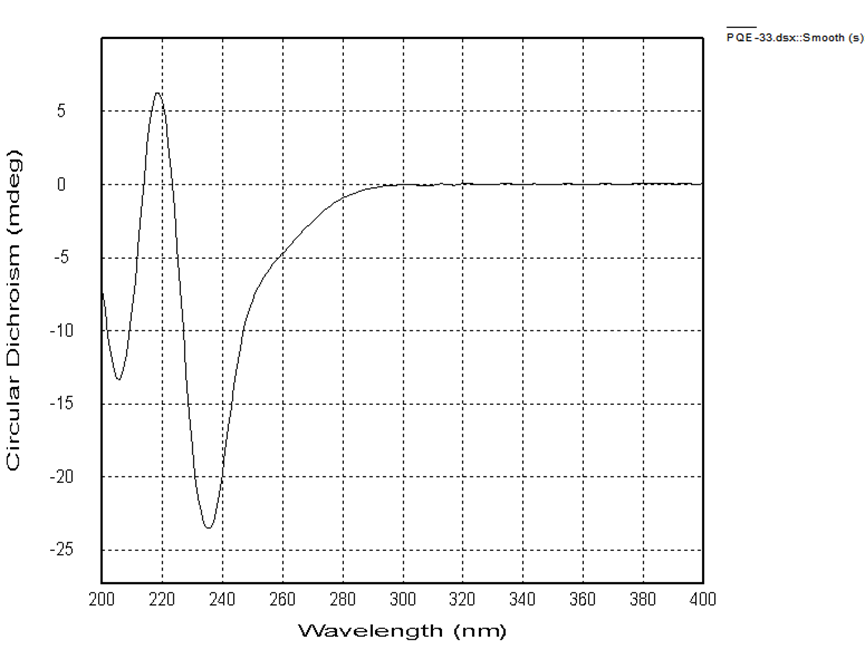


# Spectra of physico-chemical properties of 4

## Figure S33. ^1^H NMR spectrum of 4.


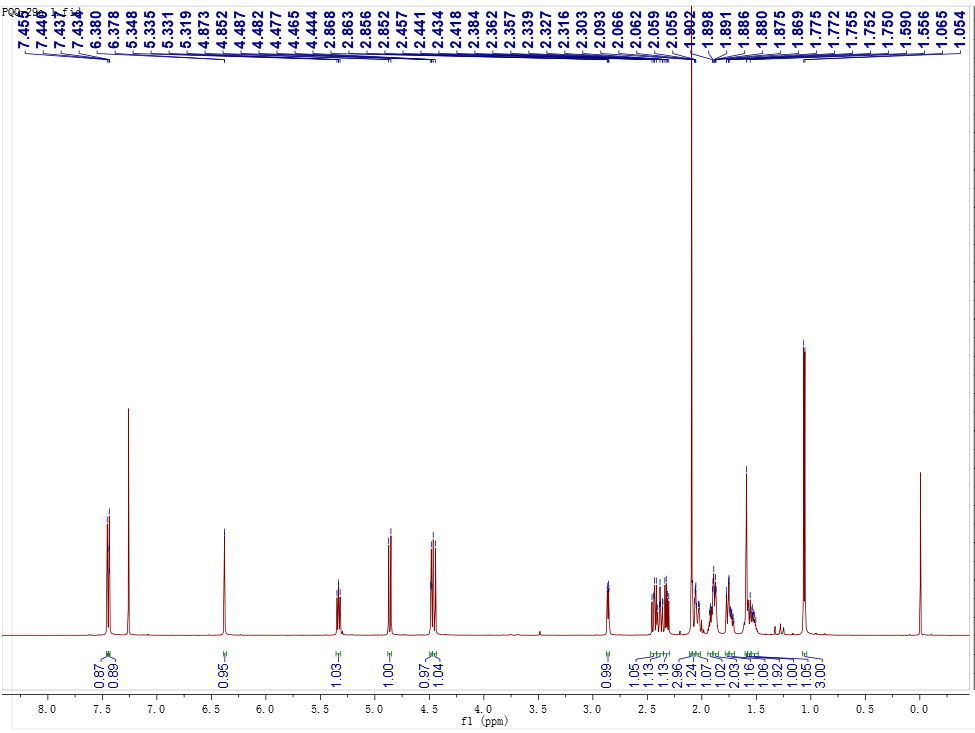


## Figure S34. ^13^C NMR spectrum of 4.


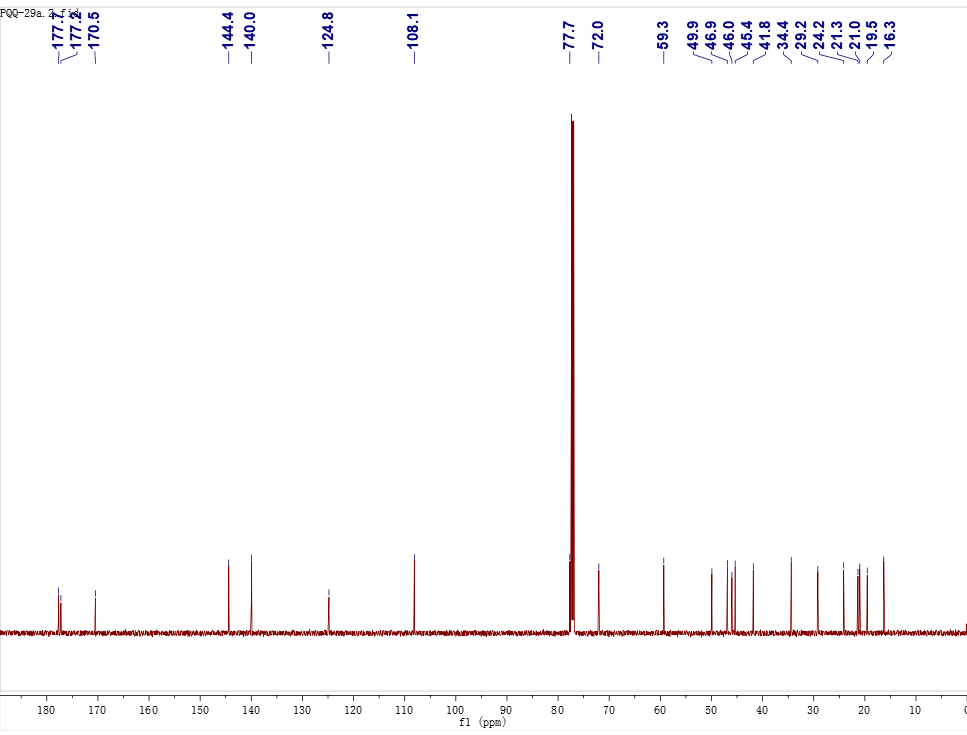


## Figure S35. 1H–1H COSY spectrum of 4.


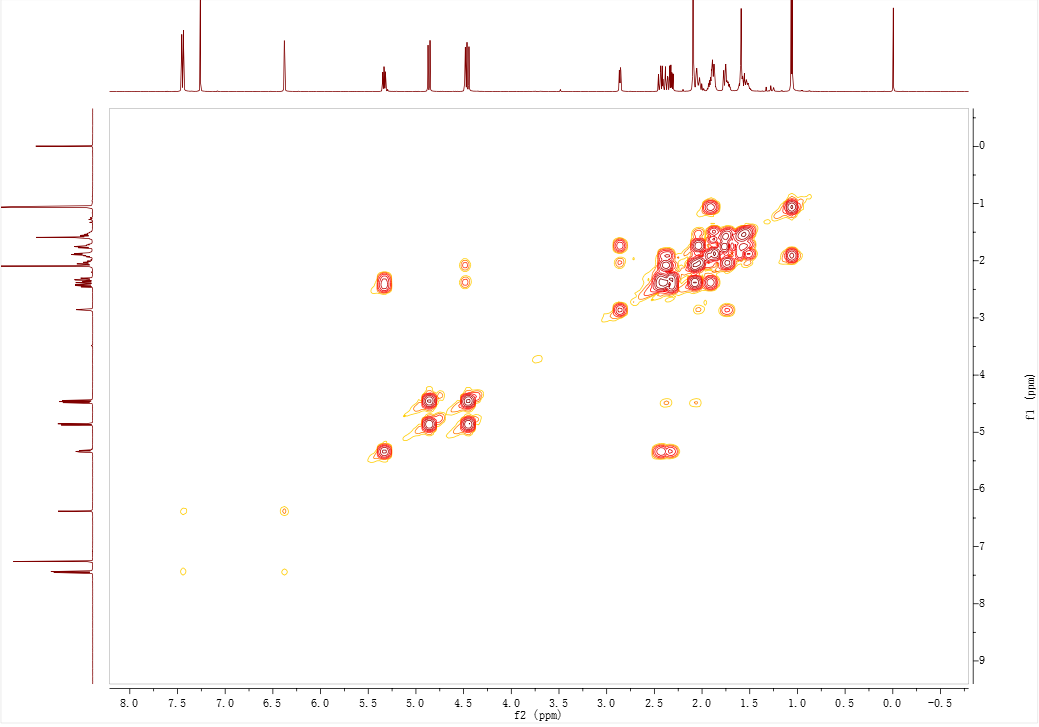


## Figure S36. HMQC spectrum of 4.


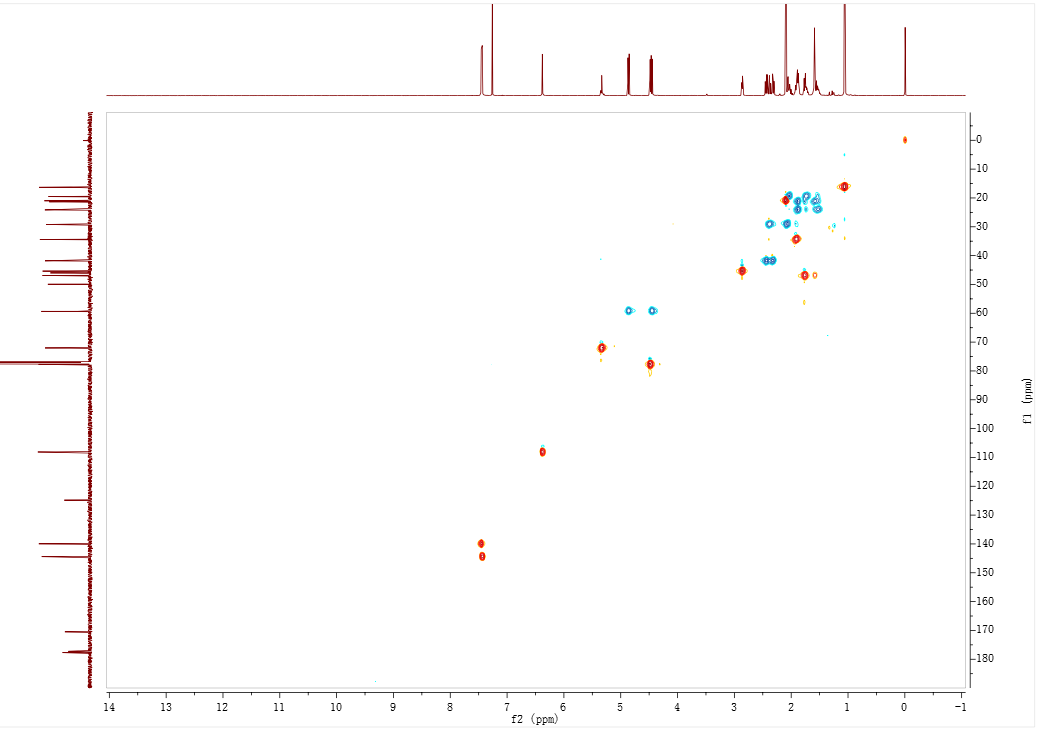


## Figure S37. HMBC spectrum of 4.


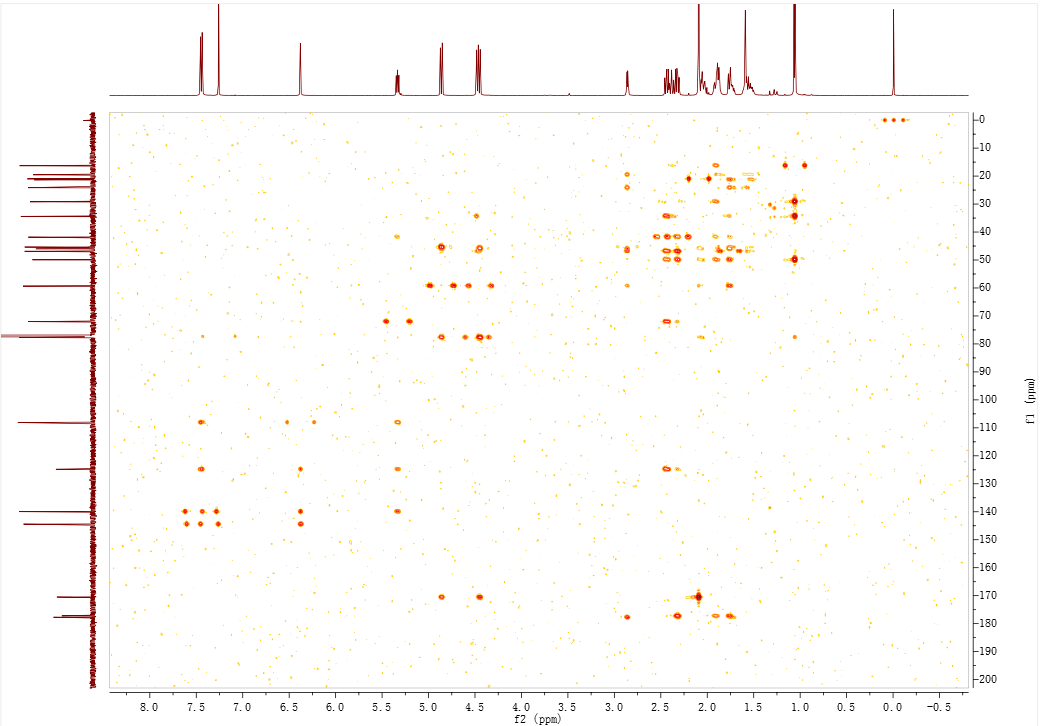


## Figure S38. NOESY spectrum of 4.


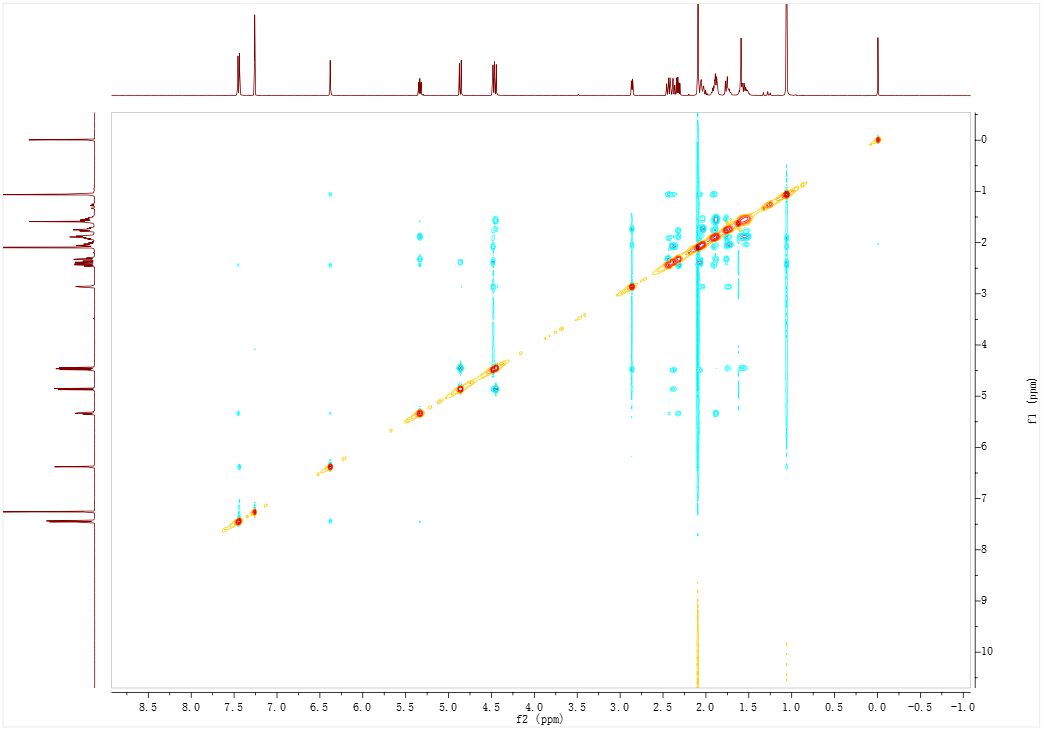


## Figure S39. HR-ESI-MS spectrum of 4.


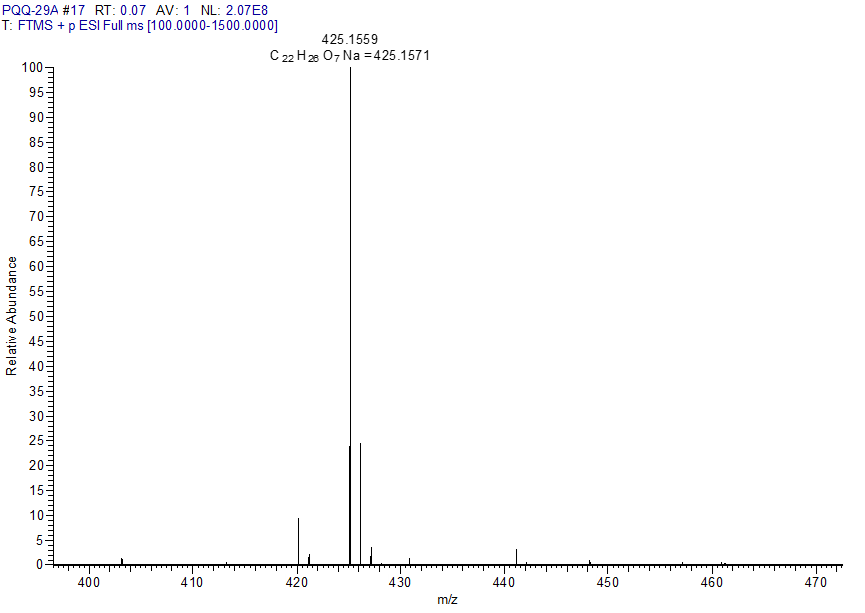


## Figure S40. UV spectrum of 4.


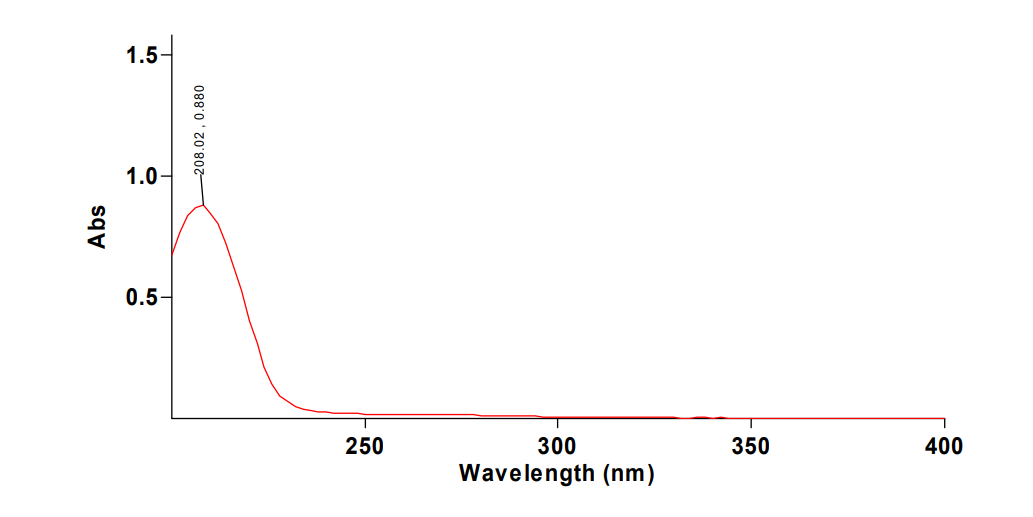


## Table S1 b3lyp/6-311+g(d,2p) optimized lowest energy 3D conformer of compound 1


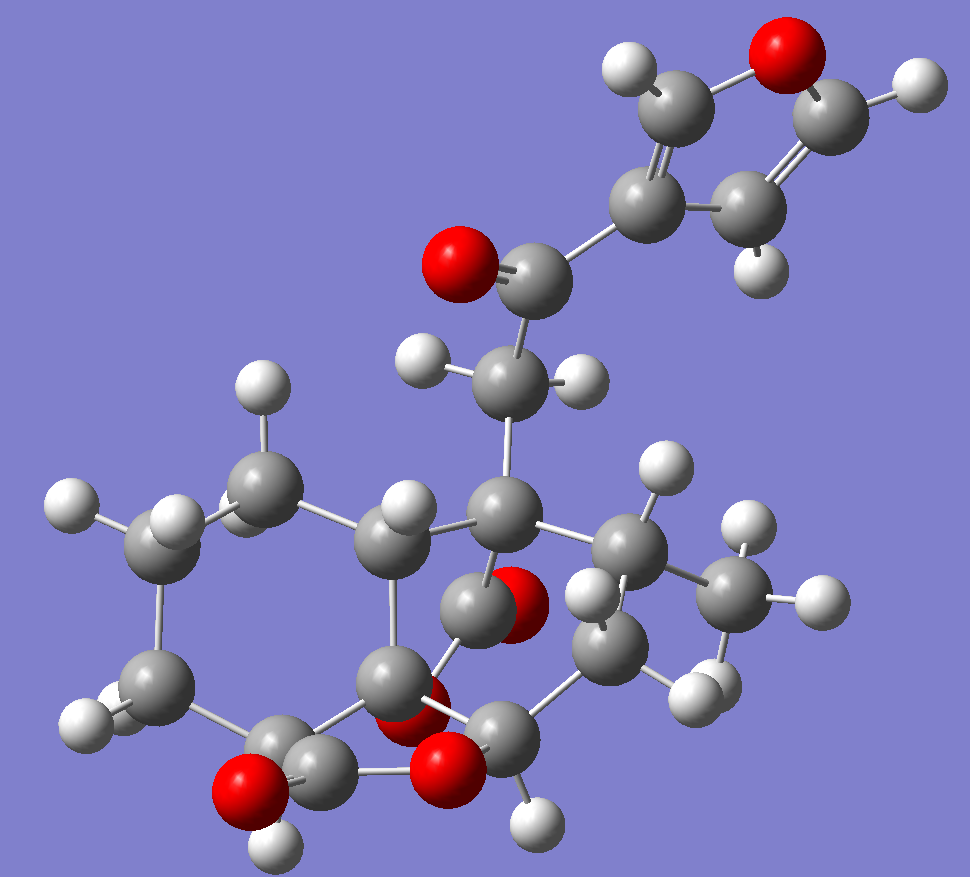


**Computational methods for ECD of compound 1**

Standard orientation:

---------------------------------------------------------------------

Center Atomic Atomic Coordinates (Angstroms)

Number Number Type X Y Z

---------------------------------------------------------------------

1 6 0 -1.740245 -2.656914 0.857219

2 6 0 -3.066451 -2.015326 1.291217

3 6 0 -3.336986 -0.680618 0.584546

4 6 0 -2.135538 0.225920 0.362665

5 6 0 -0.787709 -0.463924 0.078749

6 6 0 -0.565855 -1.673473 0.998216

7 6 0 -2.559753 1.097401 -0.836813

8 6 0 -1.375676 1.508352 -1.717585

9 6 0 -0.064324 1.780542 -0.923431

10 6 0 0.128761 0.770279 0.287372

11 6 0 -3.895346 -0.803805 -0.836013

12 8 0 -3.463297 0.251036 -1.592352

13 8 0 -4.618104 -1.658309 -1.274986

14 6 0 0.057852 3.258322 -0.511302

15 6 0 1.609937 0.505618 0.576279

16 6 0 2.366087 -0.263220 -0.499627

17 6 0 3.814498 -0.453423 -0.285723

18 8 0 1.813259 -0.721474 -1.493774

19 6 0 -0.535526 1.377467 1.531761

20 8 0 -0.036239 2.029153 2.412424

21 8 0 -1.867035 1.068132 1.513710

22 6 0 4.634756 -1.131888 -1.150538

23 8 0 5.904538 -1.143951 -0.695216

24 6 0 5.913934 -0.458873 0.493274

25 6 0 4.667702 -0.014679 0.793285

26 1 0 -0.737661 -0.818643 -0.952449

27 1 0 -1.555063 -3.554126 1.459072

28 1 0 -1.812529 -2.990823 -0.187292

29 1 0 -3.907213 -2.688628 1.092538

30 1 0 -3.041320 -1.834693 2.372998

31 1 0 -4.088784 -0.108915 1.149071

32 1 0 0.374910 -2.166279 0.728852

33 1 0 -0.477980 -1.359515 2.048268

34 1 0 -3.129061 1.959840 -0.471579

35 1 0 -1.632979 2.387718 -2.317538

36 1 0 -1.206307 0.692449 -2.426465

37 1 0 0.753723 1.563222 -1.615828

38 1 0 0.965450 3.449071 0.070610

39 1 0 0.095493 3.888422 -1.406810

40 1 0 -0.791267 3.593317 0.095180

41 1 0 2.121259 1.459194 0.761744

42 1 0 1.718390 -0.048823 1.518623

43 1 0 4.447054 -1.632598 -2.087540

44 1 0 6.873959 -0.385750 0.980157

45 1 0 4.384236 0.552318 1.668647

---------------------------------------------------------------------

## Table S2 b3lyp/6-311+g(d,2p) optimized lowest energy 3D conformer of compound 2


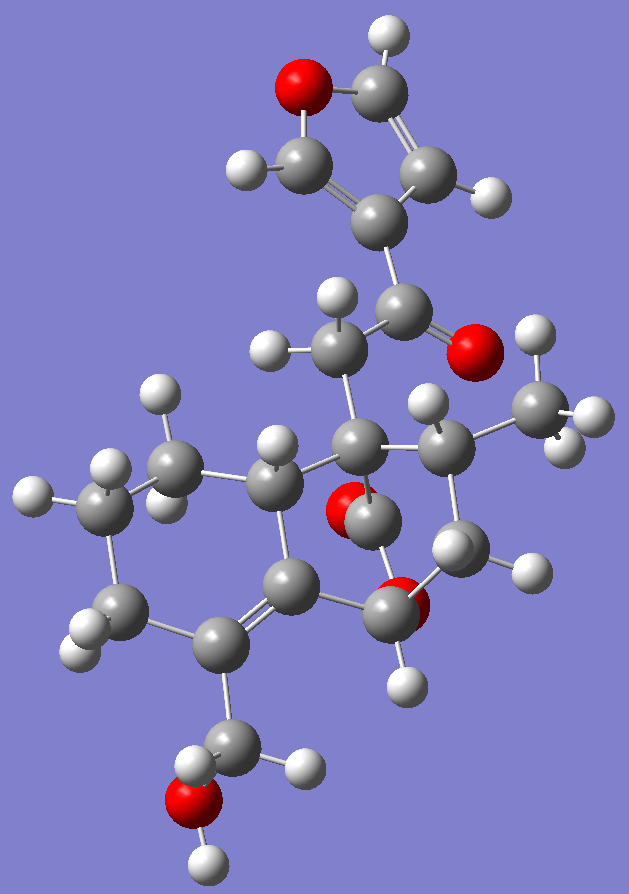


**Computational methods for ECD of compound 2**

Standard orientation:

---------------------------------------------------------------------

Center Atomic Atomic Coordinates (Angstroms)

Number Number Type X Y Z

---------------------------------------------------------------------

1 6 0 -1.831555 -2.666006 -0.924814

2 6 0 -3.271115 -2.142877 -0.811494

3 6 0 -3.396063 -0.790068 -0.134830

4 6 0 -2.313130 -0.055661 0.176015

5 6 0 -0.888575 -0.523131 -0.078010

6 6 0 -0.832736 -1.538801 -1.225953

7 6 0 -2.315337 1.378723 0.644666

8 6 0 -1.963752 2.277186 -0.548744

9 6 0 -0.649977 1.762292 -1.202913

10 6 0 0.029626 0.748459 -0.202926

11 6 0 -4.822436 -0.351746 0.113369

12 8 0 -5.530708 -1.270144 0.950781

13 6 0 0.232879 2.940355 -1.636147

14 6 0 1.484488 0.447680 -0.587220

15 6 0 2.251986 -0.546792 0.282992

16 6 0 3.721322 -0.569867 0.109803

17 8 0 1.713036 -1.323172 1.060276

18 6 0 4.585388 0.207721 -0.745996

19 6 0 5.847839 -0.229123 -0.508382

20 8 0 5.839590 -1.224053 0.434856

21 6 0 4.552684 -1.416989 0.795432

22 6 0 -0.008303 1.401106 1.181146

23 8 0 0.946748 1.735271 1.841093

24 8 0 -1.274476 1.587827 1.645854

25 1 0 -0.533085 -1.051431 0.813233

26 1 0 -1.536988 -3.145178 0.018137

27 1 0 -1.780181 -3.441992 -1.697811

28 1 0 -3.904333 -2.867086 -0.285370

29 1 0 -3.715408 -2.050013 -1.816351

30 1 0 -1.083465 -1.065038 -2.185766

31 1 0 0.176697 -1.953976 -1.316037

32 1 0 -3.233010 1.694413 1.139352

33 1 0 -2.787697 2.287410 -1.270036

34 1 0 -1.833093 3.301795 -0.178336

35 1 0 -0.906013 1.183824 -2.099207

36 1 0 -4.877436 0.665757 0.522961

37 1 0 -5.383421 -0.348982 -0.829345

38 1 0 -5.047743 -1.314962 1.791651

39 1 0 -0.354235 3.641981 -2.240187

40 1 0 0.618063 3.491329 -0.769314

41 1 0 1.085561 2.622190 -2.244246

42 1 0 1.528199 0.073929 -1.621097

43 1 0 2.052194 1.382478 -0.580116

44 1 0 4.300380 0.985932 -1.439419

45 1 0 6.819341 0.038537 -0.894061

46 1 0 4.362750 -2.171504 1.542531

---------------------------------------------------------------------

## Table S3 b3lyp/6-311+g(d,2p) optimized lowest energy 3D conformer of compound 3


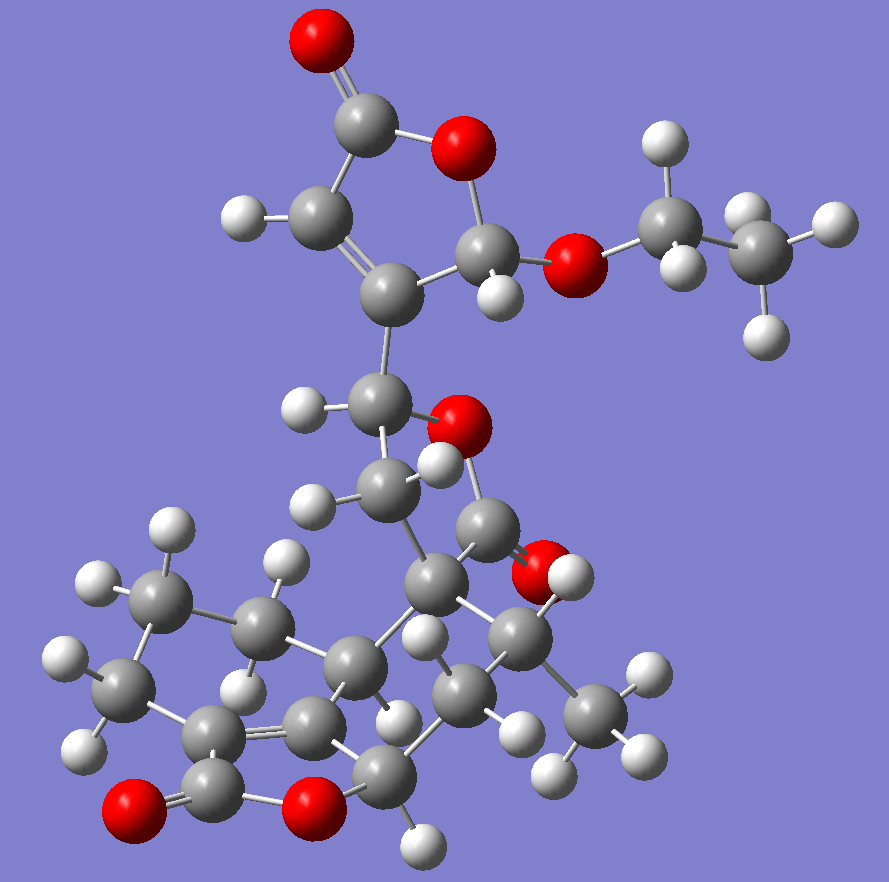


**Computational methods for ECD of compound 3**

Standard orientation:

---------------------------------------------------------------------

Center Atomic Atomic Coordinates (Angstroms)

Number Number Type X Y Z

---------------------------------------------------------------------

1 6 0 -2.522130 2.381331 1.880087

2 6 0 -3.993237 2.026096 1.585638

3 6 0 -4.056179 1.087157 0.423821

4 6 0 -3.000927 0.500676 -0.153874

5 6 0 -1.574615 0.617653 0.296804

6 6 0 -1.579994 1.167002 1.745188

7 6 0 -3.465244 -0.469643 -1.193659

8 6 0 -2.992007 -1.897935 -0.843121

9 6 0 -1.844222 -1.950484 0.206949

10 6 0 -0.827319 -0.772344 0.077012

11 6 0 -5.284787 0.571080 -0.225831

12 8 0 -4.902363 -0.351060 -1.177106

13 8 0 -6.439432 0.845994 -0.007666

14 6 0 -0.172735 -0.698605 -1.316735

15 8 0 1.126550 -0.294804 -1.203962

16 6 0 1.485105 -0.104881 0.192718

17 6 0 0.429464 -0.899944 0.966041

18 8 0 -0.667038 -0.907058 -2.396073

19 6 0 -1.208721 -3.349106 0.162607

20 6 0 3.481717 -1.296601 1.287719

21 6 0 4.954390 -1.302577 1.074437

22 8 0 5.220450 -0.504160 -0.015311

23 6 0 3.997367 0.066511 -0.514891

24 6 0 2.911707 -0.512115 0.371742

25 8 0 5.810005 -1.882625 1.688414

26 8 0 3.979350 1.448310 -0.401356

27 6 0 4.870950 2.129765 -1.301236

28 6 0 4.654194 3.621983 -1.131947

29 1 0 -1.070605 1.369857 -0.330548

30 1 0 -2.191317 3.162578 1.182227

31 1 0 -2.431647 2.804109 2.886923

32 1 0 -4.463241 1.553884 2.460243

33 1 0 -4.582676 2.929562 1.386512

34 1 0 -1.901546 0.376929 2.438018

35 1 0 -0.569498 1.466258 2.044823

36 1 0 -3.126530 -0.199459 -2.198590

37 1 0 -2.678101 -2.409168 -1.755918

38 1 0 -3.849699 -2.443672 -0.436569

39 1 0 -2.303101 -1.823427 1.198077

40 1 0 1.414634 0.972228 0.396279

41 1 0 0.297240 -0.541393 1.987742

42 1 0 0.746679 -1.944541 1.015486

43 1 0 -0.566210 -3.555245 1.024211

44 1 0 -1.996638 -4.110145 0.162474

45 1 0 -0.619523 -3.490288 -0.751582

46 1 0 3.021429 -1.853006 2.093840

47 1 0 3.907138 -0.248853 -1.563772

48 1 0 5.904622 1.848561 -1.067287

49 1 0 4.654005 1.810673 -2.331853

50 1 0 5.320685 4.175975 -1.801539

51 1 0 3.620526 3.893782 -1.368850

52 1 0 4.866549 3.927906 -0.102676

---------------------------------------------------------------------

## Table S4 Crystal data and structure refinement for 4.

| Identification code | **4** |
| --- | --- |
| Empirical formula | C_22_H_26_O_7_ |
| Formula weight | 402.43 |
| Temperature/K | 100.00(10) |
| Crystal system | orthorhombic |
| Space group | P2_1_2_1_2_1_ |
| a/Å | 6.70000(10) |
| b/Å | 12.87640(10) |
| c/Å | 22.6225(2) |
| α/° | 90 |
| β/° | 90 |
| γ/° | 90 |
| Volume/Å^3^ | 1951.69(4) |
| Z | 4 |
| ρ_calc_g/cm^3^ | 1.370 |
| μ/mm^‑1^ | 0.845 |
| F(000) | 856.0 |
| Crystal size/mm^3^ | 0.11 × 0.1 × 0.08 |
| Radiation | Cu Kα (λ = 1.54184) |
| 2Θ range for data collection/° | 7.816 to 148.718 |
| Index ranges | -7 ≤ h ≤ 8, -15 ≤ k ≤ 14, -27 ≤ l ≤ 24 |
| Reflections collected | 10153 |
| Independent reflections | 3874 [R_int_ = 0.0255, R_sigma_ = 0.0302] |
| Data/restraints/parameters | 3874/0/264 |
| Goodness-of-fit on F^2^ | 1.070 |
| Final R indexes [I>=2σ (I)] | R_1_ = 0.0264, wR_2_ = 0.0643 |
| Final R indexes [all data] | R_1_ = 0.0276, wR_2_ = 0.0650 |
| Largest diff. peak/hole / e Å^-3^ | 0.17/-0.16 |
| Flack parameter | 0.07(7) |

## Table S5  Crystal data and structure refinement for 10.

| Identification code | **10** |
| --- | --- |
| Empirical formula | C_22_H_26_O_7_ |
| Formula weight | 402.43 |
| Temperature/K | 99.99(10) |
| Crystal system | orthorhombic |
| Space group | P2_1_2_1_2_1_ |
| a/Å | 6.56600(10) |
| b/Å | 13.2097(2) |
| c/Å | 22.6129(3) |
| α/° | 90 |
| β/° | 90 |
| γ/° | 90 |
| Volume/Å^3^ | 1961.33(5) |
| Z | 4 |
| ρ_calc_g/cm^3^ | 1.363 |
| μ/mm^‑1^ | 0.841 |
| F(000) | 856.0 |
| Crystal size/mm^3^ | 0.11 × 0.08 × 0.06 |
| Radiation | Cu Kα (λ = 1.54184) |
| 2Θ range for data collection/° | 7.75 to 148.186 |
| Index ranges | -8 ≤ h ≤ 8, -14 ≤ k ≤ 15, -28 ≤ l ≤ 27 |
| Reflections collected | 18805 |
| Independent reflections | 3897 [R_int_ = 0.0619, R_sigma_ = 0.0489] |
| Data/restraints/parameters | 3897/0/264 |
| Goodness-of-fit on F^2^ | 1.047 |
| Final R indexes [I>=2σ (I)] | R_1_ = 0.0332, wR_2_ = 0.0800 |
| Final R indexes [all data] | R_1_ = 0.0475, wR_2_ = 0.0820 |
| Largest diff. peak/hole / e Å^-3^ | 0.16/-0.16 |
| Flack parameter | 0.04(9) |
